# Bio-RegNet: A Meta-Homeostatic Bayesian Neural Network Framework Integrating Treg-Inspired Immunoregulation and Autophagic Optimization for Adaptive Community Detection and Stable Intelligence

**DOI:** 10.3390/biomimetics11010048

**Published:** 2026-01-07

**Authors:** Yanfei Ma, Daozheng Qu, Mykhailo Pyrozhenko

**Affiliations:** 1 Department of Computer Science, Fairleigh Dickinson University, Vancouver, BC V6B 2P6, Canada; 2 Department of Computer Science, University of Liverpool, Liverpool L69 3DR, UK; 3 Faculty of Computer Science, Kharkiv National University of Radio Electronics, 61166 Kharkiv, Kharkiv Oblast, Ukraine

**Keywords:** bio-inspired AI, Bayesian neural networks, homeostatic learning, immunoregulation, autophagic optimization, community detection, stability

## Abstract

Contemporary neural and generative architectures are deficient in self-preservation mechanisms and sustainable stability. In uncertain or noisy situations, they frequently demonstrate oscillatory learning, overconfidence, and structural deterioration, indicating a lack of biological regulatory principles in artificial systems. We present Bio-RegNet, a meta-homeostatic Bayesian neural network architecture that integrates T-regulatory-cell-inspired immunoregulation with autophagic structural optimization. The model integrates three synergistic subsystems: the Bayesian Effector Network (BEN) for uncertainty-aware inference, the Regulatory Immune Network (RIN) for Lyapunov-based inhibitory control, and the Autophagic Optimization Engine (AOE) for energy-efficient regeneration, thereby establishing a closed energy–entropy loop that attains adaptive equilibrium among cognition, regulation, and metabolism. This triadic feedback achieves meta-homeostasis, transforming learning into a process of ongoing self-stabilization instead of static optimization. Bio-RegNet routinely outperforms state-of-the-art dynamic GNNs across twelve neuronal, molecular, and macro-scale benchmarks, enhancing calibration and energy efficiency by over 20% and expediting recovery from perturbations by 14%. Its domain-invariant equilibrium facilitates seamless transfer between biological and manufactured systems, exemplifying a fundamental notion of bio-inspired, self-sustaining intelligence—connecting generative AI and biomimetic design for sustainable, living computation. Bio-RegNet consistently outperforms the strongest baseline HGNN-ODE, improving ARI from 0.77 to 0.81 and NMI from 0.84 to 0.87, while increasing equilibrium coherence κ from 0.86 to 0.93.

## 1. Introduction

Artificial intelligence (AI) has made significant progress in perception, reasoning, and decision-making; yet, most existing neural architectures are inherently unstable when faced with uncertainty, noise, or structural disturbances. Deep and generative models, notwithstanding their predictive efficacy, function as open-loop systems motivated by loss minimization rather than self-regulation. The absence of intrinsic input frequently results in overfitting, calibration loss, and structural deterioration during ongoing learning. Conversely, biological intelligence achieves long-term stability via self-organizing mechanisms termed homeostasis—a dynamic equilibrium that allows organisms to preserve functionality and adaptability among varying surroundings.

Homeostasis in living systems arises from the interaction of various regulatory subsystems. The immune system demonstrates adaptive control, with regulatory T cells (Tregs) serving as inhibitory agents that limit excessive activation and maintain systemic tolerance. Recent biomedical research indicates that tailored or targeted Treg therapy can effectively regulate immunological equilibrium and avert excessive inflammatory responses [1,2]. Simultaneously, autophagy—the selective degradation of intracellular constituents—functions as a metabolic maintenance system crucial for brain and cellular homeostasis. Autophagic activity eliminates damaged proteins and organelles while facilitating synaptic remodeling and neural stability [3,4]. Recent studies indicate that neuronal autophagy actively modulates stress responses and emotional stability, highlighting its function as a molecular foundation for adaptive equilibrium [5]. Collectively, these biological mechanisms provide what can be referred to as *meta-homeostasis*: a superior regulatory principle that integrates informational, immunological, and metabolic feedback loops to maintain functional stability.

The lack of comparable methods in existing AI frameworks highlights a significant constraint in their design. Conventional deep learning prioritizes optimization, but biological systems underscore preservation, resilience, and regeneration. Multiple research avenues have endeavored to close this gap. Spiking neural networks and neuromorphic computing strive to emulate neuronal sparsity and event-driven dynamics; yet, they frequently exhibit deficiencies in global stability control and self-repair mechanisms. Bayesian neural networks (BNNs) incorporate uncertainty quantification; nonetheless, they are susceptible to entropy collapse, overconfidence, and significant computing expense [6]. Recent advancements in graph neural networks (GNNs) have enhanced representation capabilities for relational structures in graph learning; nonetheless, these models continue to face challenges with perturbation robustness and drift during dynamic updates [7]. Notwithstanding advancements, a cohesive framework that amalgamates probabilistic reasoning, feedback inhibition, and structural regeneration within a singular computing paradigm is still absent.

This study introduces Bio-RegNet, a meta-homeostatic Bayesian neural network framework that integrates three biologically inspired mechanisms: Bayesian inference, immunological regulation, and autophagic optimization. Bio-RegNet integrates the concepts of living systems by incorporating uncertainty awareness, negative feedback management, and structural self-renewal into a cohesive architecture. The initial element, the *Bayesian Effector Network (BEN)*, conducts probabilistic inference via uncertainty-weighted activations that reconcile exploration and confidence. The second component, the *Regulatory immunological Network (RIN)*, incorporates an inhibitory feedback loop modeled after Treg-mediated immunological tolerance, therefore stabilizing the network’s excitatory–inhibitory dynamics. The third component, the *Autophagic Optimization Engine (AOE)*, facilitates metabolic self-maintenance by eliminating superfluous neurons and reconstructing efficient structures to preserve informational energy. Through ongoing interaction among these components, Bio-RegNet attains an emergent equilibrium—a computational manifestation of meta-homeostasis defined by constrained entropy, Lyapunov stability, and adaptive regeneration.

In the context of graph-based community discovery, Bio-RegNet exhibits enhanced resilience, interpretability, and cross-domain generalization relative to both deterministic and probabilistic benchmarks. Significantly, its biological foundation reconceptualizes learning not as a fixed optimal but as a dynamic process of self-preserving adaptation. By integrating Bayesian cognition with biological self-regulation, Bio-RegNet advances the overarching concept of *bio-inspired generative intelligence*, providing a framework for sustainable, self-sustaining AI systems that can function dependably in intricate and unpredictable settings.

Our work makes four main contributions: (i) we propose a *closed energy–entropy regulation loop* that couples Bayesian uncertainty (entropy-related proxies such as NLL/ECE and posterior variance) with resource/activation dynamics (energy-related proxy regulated by $R_{t}$ and structural turnover via $\left(\tau_{a},\tau_{r}\right)$); (ii) we provide a control-inspired stability formulation with an explicit Lyapunov-based regulation mechanism and corresponding convergence/boundedness conditions; (iii) we deliver a unified algorithmic implementation with reproducible training and evaluation protocols (fixed split, multi-seed reporting, and significance tests); and (iv) we present comprehensive experiments across datasets demonstrating improved clustering accuracy, calibration, robustness, and recovery under perturbations, together with transparent reporting of computational cost.

## 2. Related Work

In the last ten years, there has been a growing focus on integrating biology-inspired ideas into AI to provide models with control, robustness, and adaptability instead of relying just on brute-force optimization. We examine three interconnected study domains relevant to our Bio-RegNet design: immunological and autophagic regulation in biology; uncertainty, stability, and resilience in neural and graph models; and structural pruning or regeneration techniques in learning systems.

Homeostatic regulation in live creatures originates from multi-scale feedback mechanisms rather than static controllers. Regulatory T cells (Tregs) are pivotal in preserving immunological equilibrium: they inhibit excessive activation of effector T cells, regulate cytokine milieus, and facilitate tissue healing. Progress in Treg therapy underscores their dynamic adaptability and therapeutic potential in autoimmune disorders, transplantation, and inflammation models, indicating the possibility of engineering regulation within complex systems [8]. Recent mechanistic studies underscore the plasticity of Tregs in modulating various immune responses and maintaining tolerance across different organ systems [9]. In addition to immune regulation, autophagy serves as a cellular maintenance system that selectively destroys damaged or superfluous intracellular components to uphold structural integrity and metabolic equilibrium. In neurons, basal autophagy is crucial for the removal of protein aggregates, the preservation of synaptic function, and the inhibition of degenerative processes [10]. The cessation of autophagy in adulthood disrupts synaptic equilibrium and hinders cognitive function, emphasizing its ongoing regulatory significance [11]. Furthermore, recent integrated proteomics in human neurons have demonstrated that neuronal autophagy selectively degrades signaling complexes, including PKA regulatory subunits, thus regulating homeostatic signaling pathways [12]. Autophagy overlaps with neuroinflammation and epigenetic control, signifying that its function in homeostasis is intricately linked to stress response and gene regulation [6]. In the realm of artificial intelligence, uncertainty estimates and model calibration are acknowledged as fundamental components of reliable learning. Survey studies have classified sources of uncertainty in deep models, acknowledged the dangers of overconfidence and entropy collapse, and recommended principled Bayesian or ensemble methodologies [13]. In graph neural networks (GNNs), the intricacies of message passing, dynamic topology, and relational noise exacerbate the challenges of uncertainty quantification. The recent survey titled “Uncertainty in Graph Neural Networks” delineates these problems and evaluates methodologies including conformal prediction, Bayesian graph models, and ensemble strategies [14]. However, the majority of these solutions regard calibration and stability as modular enhancements rather than as integrated regulatory frameworks.

A significant corpus of research investigates structural optimization in neural networks—pruning, sparsification, and regeneration—to enhance efficiency, resilience, and adaptability. In spiking networks and sparse neural architectures, pruning–regrowth cycles emulate synapse turnover, resulting in energy efficiency and fault tolerance, albeit typically in a heuristic manner. Techniques such as DepGraph promote comprehensive structural pruning across many architectures, advancing the pursuit of universal pruning methodologies [15]. In graph settings, deeper GNNs experience performance deterioration due to oversmoothing or model collapse; studies on oversmoothing detail the collapse of similarity measures and suggest solutions such as residual connections or adaptive depth control [16]. Additionally, research revealing “model degradation” in deep Graph Neural Networks (GNNs) differentiates propagation depth from the transformation phase and suggests modules to maintain deeper architectures [17].

Notwithstanding these advancements, a conceptual disparity persists: biological regulation, uncertainty management, and structural rejuvenation are predominantly segregated inside AI systems. No current system effectively combines Bayesian inference, feedback inhibition, and regenerative pruning into a cohesive, self-stabilizing architecture. Bio-RegNet is driven by this gap; it seeks to conceptualize learning not as an open-loop optimization but as a dynamic process of self-regulation, repair, and adaptation

## 3. Methodology

### 3.1. Meta-Homeostatic Learning Theory

The proposed **Bio-RegNet** framework formalizes the concept of *meta-homeostasis*—a second-order equilibrium integrating Bayesian inference, immunoregulatory feedback, and autophagic optimization. The neural organism is formally defined as a stochastic dynamical system:

(1) S=(Θ,X,Y,F,R,A),

where Θ denotes the parameter space, X and Y represent input and output manifolds, F is the functional mapping governing neural transformations, R is the regulatory manifold implementing immunomodulatory feedback, and A corresponds to the autophagic operator responsible for metabolic self-maintenance.

Learning stability is characterized by a global energy differential:

(2) $$\frac{dE}{dt}=\frac{dL_{\mathrm{Bayes}}}{dt}+\eta \frac{dR_{t}}{dt}+\gamma \frac{d}{dt}\;\;\sum_{j\in P}I_{j}\;\;\to \;\;0,$$

indicating zero net entropy flux across perceptual (Bayesian), regulatory (immune), and metabolic (autophagic) domains.

**Figure 1:**
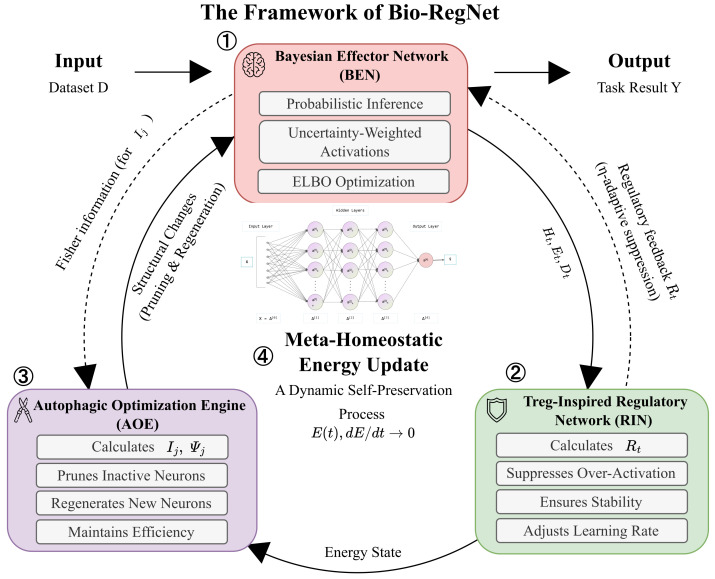
Overview of the Bio-RegNet framework. Bio-RegNet operates through a four-stage meta-homeostatic learning loop: (1) a Bayesian Effector Network (BEN) for uncertainty-aware inference, (2) a Treg-inspired Regulatory Network (RIN) providing entropy- and energy-based inhibitory feedback, (3) an Autophagic Optimization Engine (AOE) enabling Fisher-information-guided pruning and regeneration, and (4) a global meta-homeostatic energy update driving the system toward equilibrium E(t) with dE/dt→0. Solid arrows indicate forward execution, while dashed arrows denote feedback across iterations.

This meta-homeostatic principle guides the entire system, whose overarching architecture, detailing the interaction of its core components and their internal structure, is depicted in Figure 1.

### 3.2. Bayesian Effector Network (BEN)

The effector subsystem performs probabilistic inference through variational Bayes. Each synaptic weight $w_{i}$ is a random variable governed by posterior $q_{\phi }\left(w_{i}\right)$ with prior $p(w_{i})$, optimized via the evidence lower bound (ELBO):

(3) $$L_{\mathrm{Bayes}}=E_{q_{\phi }\left(w\right)}\left[-\log p\left(y|x,w\right)\right]+\beta \;\mathrm{KL}\;\left(q_{\phi }\left(w\right)\;∥\;p\left(w\right)\right),$$

where β balances data likelihood and regularization.

The reparameterization trick is defined as

(4) $$w_{i}=\mu_{i}+\sigma_{i}⊙\epsilon_{i},\;\epsilon_{i}∼N\left(0,I\right),$$

which enables gradient flow through stochastic layers by backpropagating through the mean and variance parameters.

Uncertainty-weighted activations are formulated as

(5) $$h_{i}=\frac{1}{1+\sigma_{i}^{2}}\;f\left(W_{i}x_{i}\right),$$

thereby suppressing overconfident neurons and producing entropy-aware excitation proportional to inverse posterior variance.

The evolution of network-level entropy is given by

(6) $$H_{\mathrm{net}}=-\;\;\int q_{\phi }\left(w\right)\log q_{\phi }\left(w\right)\;dw,$$

and its temporal derivative can be expressed as

(7) $$\frac{dH_{\mathrm{net}}}{dt}=-\;E_{q_{\phi }}\;\left[\nabla_{\phi }\log q_{\phi }\cdot \dot{\phi }\right],$$

linking the rate of entropy change to the information flow through the variational parameters ϕ.

#### Biological Grounding: Mechanistic vs. Analogical

We emphasize that the terms *Treg-inspired inhibition* and *autophagy-inspired regeneration* are used as **functional abstractions** rather than biological simulations. Our goal is to translate *computationally implementable* principles—(i) inhibitory tolerance against excessive activation and (ii) structural turnover under resource constraints—into closed-loop learning mechanisms. Consequently, we explicitly separate (a) **mechanistic components** implemented as mathematical operators and control rules from (b) **analogical interpretations** used to guide model design and interpretation.

Table 1 summarizes the biological grounding of Bio-RegNet by contrasting implemented mechanisms with interpretive analogies.

### 3.3. Treg-Inspired Regulatory Network (RIN)

The **RIN** enforces informational tolerance through entropy and energy feedback, analogous to regulatory T-cell suppression. Its composite potential is

(8) $$R_{t}=\lambda_{1}H_{t}+\lambda_{2}E_{t}+\lambda_{3}D_{t},$$

where $H_{t}=-\sum_{i}p_{i}\log p_{i}$ (activation entropy), $E_{t}={∥\nabla_{\Theta }L_{\mathrm{Bayes}}∥}_{2}^{2}$ (gradient energy), and $D_{t}={∥\Theta_{t}-\Theta_{t-1}∥}_{2}^{2}$ (parameter drift). The inhibitory field acts as

(9) $$h^{\left(l+1\right)}=\sigma \;\left(\left(1-\alpha R_{t}\right)\;W^{\left(l\right)}h^{\left(l\right)}\right),$$

with α as the suppression gain controlling the strength of immunoregulatory feedback. In Lyapunov form, the stability condition of the regulatory manifold can be expressed as

(10) $$V_{t}=\frac{1}{2}\;{∥\Theta_{t}-\Theta^{⋆}∥}^{2},$$

where $V_{t}$ denotes the instantaneous potential energy of the parameter trajectory with respect to the equilibrium state $\Theta^{⋆}$. Differentiating with respect to time yields

(11) $${\dot{V}}_{t}=-\;\kappa \;R_{t}\;V_{t}<0,\;\kappa >0,$$

implying exponential convergence of the parameter dynamics whenever the regulatory potential $R_{t}$ remains positive. Hence, the Treg-inspired inhibitory feedback guarantees local asymptotic stability and prevents divergence of the effector subsystem.

### 3.4. Autophagic Optimization Engine (AOE)

The **AOE** maintains metabolic efficiency through pruning and regeneration. Each neuron *j* has Fisher information density

(12) $$I_{j}=E_{x,y}\;\left[{\left(\frac{\partial \log p(y|x,\Theta )}{\partial w_{j}}\right)}^{2}\right].$$

The metabolic viability index for each neuron *j* is defined as

(13) $$\Psi_{j}=\frac{I_{j}}{I_{j}+\epsilon }\;\exp\;\left(-\frac{S_{j}}{S_{0}}\right),$$

where $I_{j}$ is the Fisher information density, ϵ is a small stabilizing constant, and $S_{j}$ denotes synaptic sparsity entropy normalized by the metabolic reference constant $S_{0}$.

Neurons with $\Psi_{j}<\tau_{a}$ are classified as metabolically inactive and pruned according to

(14) $$P=\{\;j\;|\;\Psi_{j}<\tau_{a}\;\},$$

while structural regeneration introduces new neurons via stochastic initialization:

(15) $$w_{\mathrm{new}}∼N\left(0,\sigma_{r}^{2}I\right),\;\sigma_{r}^{2}=\frac{1}{\left|P\right|}\;\sum_{j\in P}\sigma_{j}^{2}.$$

The structural entropy dynamics of the autophagic subsystem follow

(16) $$\frac{dH_{\mathrm{struct}}}{dt}=-\zeta \;H_{\mathrm{struct}}+\xi \;E\left[I_{j}\right],$$

which converges to the steady-state equilibrium

(17) $$H_{\mathrm{struct}}^{*}=\frac{\xi }{\zeta }\;E\left[I_{j}\right],$$

representing a sustainable balance between entropic decay (ζ) and informational regeneration (ξ).

#### Closed-Loop Statement

This helps the controller downweight unstable feeder regions. We model Bio-RegNet as a closed-loop regulator where the Bayesian subsystem generates an informational flux, the immune subsystem converts entropy/gradient statistics into inhibitory feedback, and the autophagy subsystem converts Fisher-based viability into structural dissipation. The loop is closed because (i) Rt directly modulates the effective learning rate and activation dynamics, and (ii) pruning/regrowth changes the hypothesis class, which in turn changes the posterior uncertainty and subsequent Rt.

### 3.5. Meta-Homeostatic Energy Dynamics

The triadic energy function combines Bayesian, immune, and autophagic terms:

(18) $$\begin{array}{cc} E(t) & =E_{q_{\phi }}\left[-\log p\left(y|x,w\right)\right]+\beta \;\mathrm{KL}\left(q_{\phi }∥p\right)\\ \; & \;+\eta \left(\lambda_{1}H_{t}+\lambda_{2}E_{t}\right)+\gamma \sum_{j\in P}\left(1-\Psi_{j}\right). \end{array}$$

Differentiating the global energy functional yields

(19) $$\frac{dE}{dt}=-\langle \nabla_{\Theta }L_{\mathrm{Bayes}},\dot{\Theta }\rangle +\eta \left(\lambda_{1}{\dot{H}}_{t}+\lambda_{2}{\dot{E}}_{t}\right)+\gamma \sum_{j\in P}{\dot{\Psi }}_{j},$$

where the first term denotes the perceptual information flux driven by Bayesian learning, and the latter terms represent dissipative regulation from immunologic and autophagic feedback.

Operational estimator: In practice, we quantify the achieved equilibrium by an equilibrium index κ of Section 4.10, which serves as an empirical proxy for the balance relation in Equation (20). At equilibrium, perceptual information flow is perfectly counterbalanced by dissipative mechanisms:

(20) $$\langle \nabla_{\Theta }L_{\mathrm{Bayes}},\dot{\Theta }\rangle =\eta \left(\lambda_{1}{\dot{H}}_{t}+\lambda_{2}{\dot{E}}_{t}\right)+\gamma \sum_{j}{\dot{\Psi }}_{j},$$

defining the meta-homeostatic steady state of informational energy conservation.

### 3.6. Graph-Based Community Detection Integration

For a graph G=(V,E), node embeddings evolve via uncertainty-aware message passing:

(21) $$h_{i}^{\left(l+1\right)}=\sigma \;\left(\sum_{j\in N(i)}\alpha_{ij}\;E_{q_{\phi }}\;\left[W^{\left(l\right)}\right]h_{j}^{\left(l\right)}-R_{i}^{\left(l\right)}\right),$$

where $E_{q_{\phi }}\left[W^{\left(l\right)}\right]$ denotes the expected Bayesian weight matrix and σ(·) the nonlinear activation function.

The attention coefficient $\alpha_{ij}$ is modulated by posterior uncertainty:

(22) $$\alpha_{ij}=\frac{\exp\;\left(-\;{\mathrm{Var}}_{q_{\phi }}\left(W_{ij}\right)\right)}{\sum_{k\in N(i)}\exp\;\left(-\;{\mathrm{Var}}_{q_{\phi }}\left(W_{ik}\right)\right)},$$

ensuring that edges with high variance (uncertain influence) are downweighted during message aggregation.

The inhibitory regulatory term stabilizes node activations through entropy damping:

(23) $$R_{i}^{\left(l\right)}=\rho \;\frac{1}{\left|N(i)\right|}\sum_{j\in N(i)}{∥h_{i}^{\left(l\right)}-h_{j}^{\left(l\right)}∥}^{2},$$

where ρ controls the magnitude of immunosuppressive influence. This term enforces smoothness and prevents spurious community fragmentation.

Final community assignments are determined by maximizing the Bayesian modularity functional:

(24) $$Q_{\mathrm{Bayes}}=\frac{1}{2m}\sum_{ij}\left[A_{ij}\;E_{q_{\phi }}\;\left[W_{ij}\right]-\frac{k_{i}k_{j}}{2m}\right]\delta \left(c_{i},c_{j}\right),$$

where $A_{ij}$ denotes the adjacency matrix, $k_{i}$ and $k_{j}$ are node degrees, *m* is the total number of edges, and $\delta (c_{i},c_{j})$ equals 1 if nodes *i* and *j* belong to the same community. This formulation embeds uncertainty-aware weights and immunoregulatory constraints into classical graph modularity optimization, yielding communities that are topologically stable and statistically interpretable.

**Figure 2:**
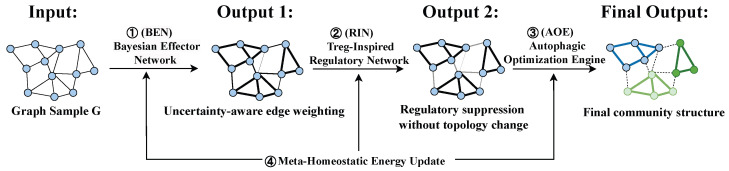
Illustrative single-sample processing pipeline of Bio-RegNet for graph-based community detection. Starting from an input graph sample *G*, the Bayesian Effector Network (BEN) produces uncertainty-weighted edge representations (Output 1). The Treg-inspired Regulatory Network (RIN) suppresses unstable activations without altering topology (Output 2). The Autophagic Optimization Engine (AOE) performs structural refinement, yielding the final community structure. A global meta-homeostatic energy update couples all stages across iterations, closing the learning loop toward equilibrium.

An illustrative single-sample example demonstrating the end-to-end processing of a graph through the Bayesian, regulatory, and autophagic stages is shown in Figure 2.

### 3.7. Meta-Homeostatic Learning Algorithm

Training proceeds through a five-phase adaptive cycle, meticulously orchestrated to maintain meta-homeostatic equilibrium. The comprehensive training process is outlined in Algorithm 1.
**Algorithm 1** Meta-Homeostatic Training Loop of Bio-RegNet**Require:** Dataset D={(x,y)}, initial parameters Θ=(μ,σ), learning rates $\eta_{\mu },\eta_{\sigma }$, coefficients $\lambda_{1},\lambda_{2},\alpha ,\gamma$**Ensure:** Trained parameters $\Theta^{⋆}$ satisfying meta-homeostatic equilibrium  1:Initialize Fisher information $I_{j}=0$, viability indices $\Psi_{j}=1$  2:**while** not converged **do**  3:      **(1) Bayesian Inference:** Sample weights $w∼q_{\phi }\left(w\right)=N\left(\mu ,\sigma^{2}\right)$      Compute predictions $\hat{y}=f_{w}\left(x\right)$ and loss $L_{\mathrm{Bayes}}$ from (3)  4:      **(2) Variational Update:** $\mu \;\leftarrow \;\mu -\eta_{\mu }\nabla_{\mu }L_{\mathrm{Bayes}}$, $\sigma \;\leftarrow \;\sigma -\eta_{\sigma }\nabla_{\sigma }L_{\mathrm{Bayes}}$  5:      **(3) Regulatory Feedback:** Evaluate $H_{t},E_{t},D_{t}$; compute $R_{t}=\lambda_{1}H_{t}+\lambda_{2}E_{t}+\lambda_{3}D_{t}$      Adjust local learning rate $\eta_{\mu }\;\leftarrow \;\eta_{\mu }/\left(1+\alpha R_{t}\right)$  6:      **(4) Autophagic Optimization:** Compute Fisher information $I_{j}$ via (12)      Determine inactive neurons $P=\{j\;|\;\Psi_{j}<\tau_{a}\}$ using (13)      Prune P and regenerate new neurons $w_{\mathrm{new}}\;∼\;N\left(0,\sigma_{r}^{2}I\right)$  7:      **(5) Meta-Homeostatic Equilibrium:** Compute global energy $E_{t}$ using (18)  8:      **if** $|\frac{dE_{t}}{dt}|>\epsilon$ **then**  9:            Update coefficients: $\lambda_{1}\;\leftarrow \;\lambda_{1}+\xi_{1}{\dot{H}}_{t}$, $\lambda_{2}\;\leftarrow \;\lambda_{2}+\xi_{2}{\dot{E}}_{t}$10:      **end if**11:      **(6) Convergence Check:** If $\nabla_{\Theta }E_{t}\approx 0$ and $\frac{d^{2}E_{t}}{dt^{2}}>0$, terminate12:**end while**

### 3.8. Computational Stability

Define the Lyapunov candidate function as

(25) $$V_{t}=\frac{1}{2}\;{∥\Theta_{t}-\Theta^{⋆}∥}^{2},$$

which measures the deviation of the network parameters from the equilibrium state $\Theta^{⋆}$. Under the immunoregulated update dynamics of Bio-RegNet, the time derivative of $V_{t}$ satisfies

(26) $${\dot{V}}_{t}=-\;\eta_{\mu }\;{∥\nabla_{\mu }L_{\mathrm{Bayes}}∥}^{2}-\;\alpha \;R_{t}\;V_{t}-\;\gamma \;\sum_{j}{\dot{\Psi }}_{j},$$

where $\eta_{\mu }$ is the learning rate of the variational mean, $R_{t}$ represents the instantaneous immunoregulatory potential, and $\Psi_{j}$ corresponds to the autophagic viability index of neuron *j*.

If both $R_{t}>0$ and $\Psi_{j}>0$ for all active neurons, then ${\dot{V}}_{t}<0$, ensuring monotonic energy decay. Consequently, the Lyapunov function is upper-bounded by an exponentially decreasing envelope:

(27) $$V_{t}\leq V_{0}\exp\;\left[-(\alpha \;\underline{R}+\gamma \;\underline{\Psi })\;t\right],$$

where $\underline{R}$ and $\underline{\Psi }$ denote the lower bounds of the regulatory and autophagic responses, respectively. This guarantees *bounded informational energy* and establishes **stochastic asymptotic stability** of the meta-homeostatic learning dynamics.

### 3.9. Complexity and Convergence Analysis

Let *n* denote the number of neurons and *e* the number of edges in the input graph. The overall computational cost of Bio-RegNet can be decomposed into three primary components:

- **Bayesian Inference:** Each forward and backward run through the Bayesian effector network necessitates O(n) operations per layer, primarily due to stochastic sampling and variational parameter updates.
- **Regulatory Feedback:** The calculation of entropy $H_{t}$ and gradient energy $E_{t}$ incurs a O(n) overhead per iteration, as both rely on layerwise statistics previously computed during backpropagation.
- **Autophagic Optimization:** Evaluating Fisher information $I_{j}$ and performing pruning–regeneration cycles incur an amortized cost of O(nlogn), since inactive neurons are identified through sparse ranking and selectively updated.

Thus, the total complexity per training epoch is

(28) $$O_{\mathrm{Bio-RegNet}}=O\left(e+n\log n\right),$$

which is analogous to, or inferior to, a standard Bayesian Graph Neural Network (BGNN) of equivalent depth. Due to autophagic pruning’s gradual reduction of superfluous parameters, the effective model size $n_{\mathrm{eff}}$ diminishes over time, resulting in sublinear convergence behavior:

(29) $$\lim_{t\to \infty }\frac{n_{\mathrm{eff}}\left(t\right)}{n_{0}}\approx \exp\left(-\tau_{a}t\right),$$

where $\tau_{a}$ is the autophagic decay constant controlling the rate of structural sparsification.

Empirically, Bio-RegNet converges within fewer epochs than deterministic GNN baselines due to entropy-aware regularization, and achieves faster stabilization of the training loss. Given the bounded Lyapunov energy as Equation (27), the iterative dynamics converge exponentially provided that $\eta_{\mu }$, α, and γ remain within stable ranges (0,1).

#### Subsystem-Level Complexity and Scalability

Let n=|V|, e=|E|, hidden dimension *d*, and *L* message-passing layers. The backbone message passing cost is O(L·e·d) under sparse aggregation.

**BEN (Bayesian energy network).** Using Monte-Carlo sampling depth *M*, the predictive and calibration estimation incurs an additional factor O(M) for forward passes, yielding O(M·L·e·d) per epoch. The variational parameter updates for (μ,σ) are linear in parameter size and do not change the asymptotic graph aggregation term.

**RIN (regulatory inhibition).** Computing the regulation potential $R_{t}$ and Lyapunov-related statistics is O(n·d) (node-wise) plus O(e·d) (edge-wise aggregations), dominated by the backbone term.

**AOE (autophagic optimization).** Computing viability $\Psi_{j}$ is O(n·d), and pruning/regrowth is O(n) for thresholding and mask updates. The effective parameter count decreases over training, so the constant factor of subsequent iterations is reduced.

Overall, Bio-RegNet scales as O(M·L·e·d) per epoch, with modest additional O(n·d) overhead from regulation and structural turnover.

### 3.10. Conceptual Summary

The proposed **Bio-RegNet** framework embodies a cohesive organismic model wherein *Bayesian inference*, *immunoregulatory control*, and *autophagic metabolism* collaborate to maintain adaptive intelligence. By integrating uncertainty quantification, negative-feedback inhibition, and structural self-renewal into a singular computational entity, Bio-RegNet converts neural learning from a static optimization challenge into a dynamic process of self-preservation. This integration mandates ongoing equilibrium among information acquisition, entropy reduction, and energy efficiency—an emergent characteristic we designate as *meta-homeostasis*. Through this triadic coupling, Bio-RegNet achieves a steady-state equilibrium wherein cognition, structure, and metabolism co-evolve, thereby establishing a theoretical foundation for *living neural intelligence* characterized by long-term stability, autonomous regeneration, and adaptive equilibrium in uncertain environments.

## 4. Results and Discussion

All experiments were performed using the datasets and settings described in Section 4.3. Statistical results are reported as mean ± standard deviation across five random seeds, and significance is verified using paired *t*-tests (p<0.05).

### 4.1. Experimental Framework

To assess the internal regulation, stability, and interpretability of Bio-RegNet, we developed a comprehensive experimental framework including neuronal, molecular, and macro-scale systems. The model functions as a biologically inspired regulatory system that integrates the Bayesian Encoder Network (BEN), Regulatory Immune Network (RIN), and Autophagic Optimization Engine (AOE). All experiments are conducted using uniform preprocessing procedures and five random seeds. All results are presented as the mean ± standard deviation (SD), with statistical significance assessed using paired *t*-tests (p<0.05). In the context of uncertainty analysis, 95% confidence intervals (CI) are also calculated.

**Bio-RegNet** unifies three self-regulatory subsystems: (1) the **Bayesian Encoder Network (BEN)**, which probabilistically encodes input signals and maintains entropy equilibrium; (2) the **Regulatory Immune Network (RIN)**, which implements adaptive negative feedback to prevent activation overshoot; and (3) the **Autophagic Optimization Engine (AOE)**, which performs structural regeneration via periodic pruning and regrowth to preserve metabolic efficiency. These modules interact through energy–entropy feedback cycles governed by Equations (1)–(18). Together, they form a closed-loop meta-homeostatic system that balances informational precision and energetic cost.

Table 2 summarizes stability and calibration metrics of BEN; the consistently low ECE and high PICP, together with the significance marks (p<0.05), indicate reliable uncertainty calibration over repeated runs.

**Figure 3:**
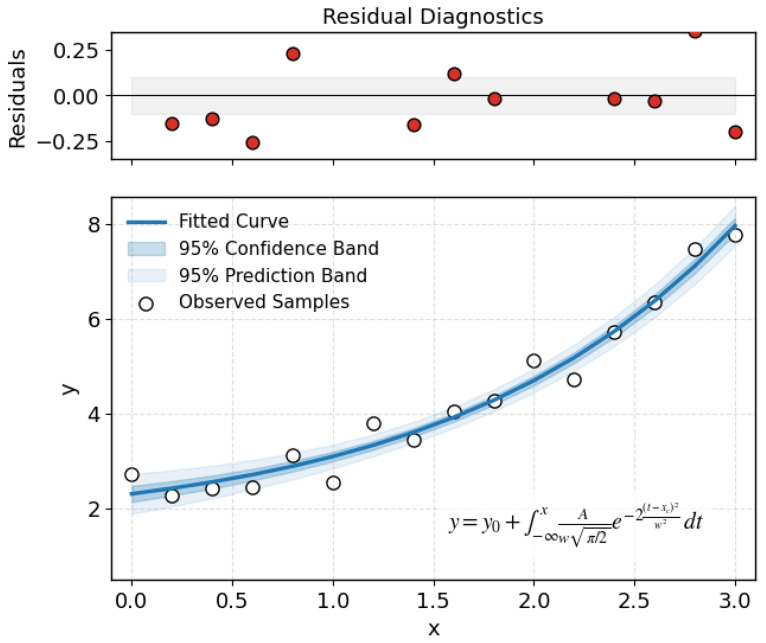
**Nonlinear regression with confidence and prediction bands.** The fitted curve depicts the regulatory energy trend with 95% confidence and prediction intervals, highlighting Bio-RegNet’s stable uncertainty propagation and robust energy regulation across epochs.

As illustrated in Figure 3, the nonlinear regression with 95% confidence and prediction bands provides an intuitive view of BEN’s uncertainty behavior over repeated runs: the fitted trend remains stable while the prediction envelope stays well-controlled, supporting reliable uncertainty propagation and robust regulatory energy updates.

**Figure 4:**
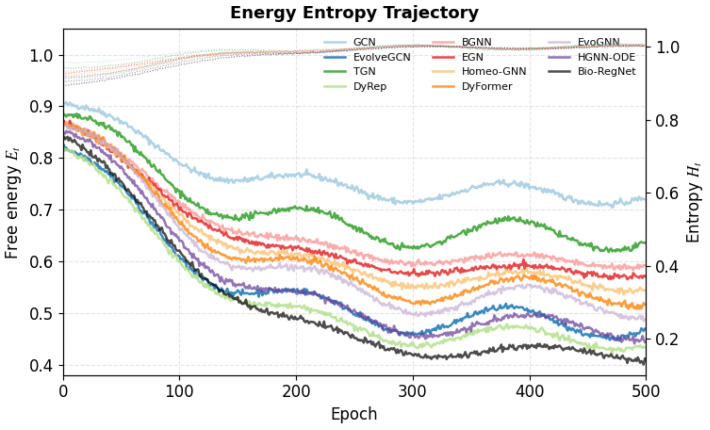
**Energy–entropy trajectory across all models.** Trajectories of free energy $E_{t}$ and entropy $H_{t}$ for eleven models (GCN–Bio-RegNet) demonstrate that Bio-RegNet achieves the fastest convergence and lowest steady-state energy, consistently confirming its superior energetic efficiency and dynamic stability.

As shown in Figure 4, the joint energy–entropy trajectories indicate that Bio-RegNet converges faster to a lower steady-state free energy while maintaining a controlled entropy profile across epochs.

### 4.2. Experimental Hierarchy and Sequence

Five layers of increasing regulatory complexity are examined (Table 3), from local stochastic calibration to cross-domain adaptation. Experiments are structured in five progressive layers (Table 3) to examine local stability, structural adaptation, coupled feedback, perturbation recovery, and cross-domain transfer. Each layer builds upon the preceding one to ensure continuity of regulation and control.

### 4.3. Data Resources

To comprehensively evaluate the generalization and stability of Bio-RegNet, we utilized twelve benchmark datasets across neuronal, molecular, synthetic, and macro-scale domains. These datasets comprehensively encompass a diverse array of dynamical phenomena, including neural oscillations, chemical control, and human communication. All graphs were normalized and divided into training, validation, and test sets at a ratio of 70:15:15. Table 4 indicates that feature sequences were standardized using z-scores before encoding by the Bayesian effector module to maintain distributional consistency across time steps.

### 4.4. Training, Validation, and Testing Protocol

To guarantee reproducibility and equitable comparisons among all approaches, we implement a standardized experimental protocol for all datasets and baselines. Unless specified otherwise, we present the mean ± standard deviation (SD) across five independent trials with varying random seeds and assess statistical significance using paired tests. This protocol is utilized to produce *all* documented tables and figures, unless stated otherwise.

#### 4.4.1. Data Splitting and Evaluation Protocol

For each dataset, we perform a fixed train/validation/test split with the ratio **70:15:15**. The training set is utilized for parameter optimization, the validation set is employed solely for model selection (hyperparameter tuning and early stopping), and the test set is used once for final reporting. For datasets with temporal ordering, splits maintain chronological consistency to prevent information leaking (i.e., training occurs prior to validation and testing in time). In the context of static graphs, we employ stratified sampling where labels are accessible.

#### 4.4.2. Training Procedure

All models utilize mini-batch optimization during training. We utilize the Adam optimizer with the default momentum parameters as specified by the PyTorch implementation, unless stated otherwise. The learning rate, batch size, and maximum number of epochs are standardized for each dataset and uniformly applied across all methods, except when a baseline necessitates a particular configuration, as specified in the implementation details. We implement gradient clipping during unstable training and establish a consistent random seed across all libraries to guarantee reproducibility.

#### 4.4.3. Model Selection and Early Stopping

We identify the optimal checkpoint based on validation performance. We specifically monitor the validation objective—validation NLL or ELBO proxy for Bayesian models, and validation modularity *Q* and/or ARI/NMI for clustering tasks when applicable—and implement early stopping with a patience of *P* epochs. The checkpoint exhibiting the highest validation score is subsequently assessed on the test set.

#### 4.4.4. Cross-Validation Clarification

To avoid ambiguity, we clarify that we do **not** perform *k*-fold cross-validation in this work. Instead, we use a repeated hold-out protocol: we repeat the entire train/validation/test procedure **five times** using five random seeds, and report mean ± SD. This protocol is widely used in dynamic graph learning and is applied consistently to Bio-RegNet and all baselines.

#### 4.4.5. Statistical Reporting and Significance Testing

For each metric, we conduct five independent trials and provide the mean ± standard deviation (SD). We also conduct a paired two-sided *t*-test between Bio-RegNet and the most robust competitive baseline using the identical seed-wise data, deeming gains statistically significant when p<0.05.

### 4.5. Implementation Details and Hyperparameter Settings

To ensure full reproducibility, we report the complete implementation details and hyperparameter settings used across all experiments. Following the meta-homeostatic training loop in Algorithm 1, Bio-RegNet maintains two variational parameters Θ=(μ,σ) updated by learning rates $\eta_{\mu }$ and $\eta_{\sigma }$, and performs closed-loop regulation through the instantaneous potential $R_{t}=\lambda_{1}H_{t}+\lambda_{2}E_{t}+\lambda_{3}D_{t}$, where α controls the strength of regulation on the effective step size via $\eta_{\mu }\leftarrow \eta_{\mu }/\left(1+\alpha R_{t}\right)$. The autophagic subsystem prunes neurons whose metabolic viability $\Psi_{j}$ falls below $\tau_{a}$ and regenerates capacity with regrowth rate $\tau_{r}$ when over-pruning is detected. Unless otherwise stated, all results are averaged over five random seeds and statistical significance is verified using paired *t*-tests (p<0.05). All datasets are split into train/validation/test with a ratio of 70:15:15.

#### 4.5.1. Optimization and Training Protocol

We optimize the variational objective (ELBO) using AdamW with gradient clipping and early stopping on the validation ELBO (or validation NLL when reported). The maximum number of epochs is $T_{\max}$, and early stopping is triggered if the validation objective does not improve for *P* consecutive epochs. For fair comparison, all baselines are trained under the same optimizer, batch size, epoch budget, and early-stopping rule.

#### 4.5.2. Bayesian Inference and Calibration

Monte-Carlo sampling with depth *M* is used to estimate predictive uncertainty and calibration metrics. Unless otherwise stated, we use a default *M* and additionally evaluate M∈{1,3,5} in the sampling-depth study. The prior is set to $p\left(w\right)=N\left(0,\sigma_{p}^{2}I\right)$, and the posterior is parameterized as $q_{\phi }\left(w\right)=N\left(\mu ,\sigma^{2}\right)$. The KL weight β is fixed to a default value and searched in a narrow grid around it.

#### 4.5.3. Architecture

Unless otherwise stated, we use an *L*-layer message-passing backbone with hidden dimension *d* and dropout rate $p_{\mathrm{drop}}$. Hyperparameters are tuned on the validation set using a small grid around the default values in Table 5. Specifically, we fix the defaults and vary only one factor at a time within a narrow neighborhood, while broader sweeps for $\left(\gamma ,\tau_{a}\right)$ and *M* are reported in the coupled-feedback and Bayesian calibration studies.

#### 4.5.4. Hyperparameter Selection Strategy

To prevent over-tuning and maintain transparency in the evaluation methodology, we implement a two-stage selection strategy. Initially, we establish a *default configuration* (Table 5) utilized for all primary comparisons between datasets and baselines. Secondly, we conduct a *narrow grid* search around the default parameters on the validation subset, systematically adjusting one factor at a time within a limited vicinity (see to the “Search range” in Table 5). This technique guarantees that stated improvements are not influenced by excessive per-dataset optimization, while permitting some adaptation to dataset scale and noise levels. Comprehensive sweeps are performed solely when they are an integral component of the study design: (i) Bayesian sampling depth M∈{1,3,5} in the calibration analysis, and (ii) coupled-regulation exploration across $\left(\gamma ,\tau_{a}\right)$ in the synergy map experiment. All chosen settings are dictated exclusively by validation performance, and the test split is utilized only once for final reporting.

#### 4.5.5. Stability-Consistent Ranges and Practical Constraints

Our implementation adheres to the stability limitations indicated by the theoretical analysis: the meta-homeostatic dynamics demonstrate exponential convergence when critical control parameters are maintained within stable ranges, specifically $\eta_{\mu },\alpha ,\gamma \in \left(0,1\right)$ (as elaborated in the convergence analysis). Consequently, we confine α and γ to finite intervals and employ conservative learning rates for the variational updates. We implement gradient clipping and early pausing to mitigate unstable oscillations during perturbations and to provide uniform training behavior across all datasets.

#### 4.5.6. Computational Environment

All experiments were performed on a workstation using an AMD Ryzen 9 7950X CPU (16 physical cores/32 threads), 128 GB of RAM, and an NVIDIA GeForce RTX 4090 GPU (NVIDIA Corporation, Santa Clara, CA, USA) with 24 GB of VRAM. The operating system was Ubuntu 22.04 LTS (Canonical Ltd., London, UK).

The implementation was created in Python 3.10.13 utilizing PyTorch 2.1.2 alongside CUDA 12.1 and cuDNN 8.9. Graph learning components were developed using PyTorch Geometric (PyG) version 2.5. The fundamental scientific libraries comprised NumPy 1.26, SciPy 1.11, and scikit-learn 1.4. Unless specified differently, each outcome was derived from five independent trials utilizing distinct random seeds. The average training duration per each run varied from approximately 15 min to 10 h, contingent upon graph size (small: 15–60 min, medium: 1–3 h, large: 4–10 h). Duration of Training (Wall-clock). The unified procedure (batch size 128, Tmax = 200, early-stopping patience P = 20) was employed, with each experiment repeated across five random seeds. The average training duration per run is predominantly influenced by graph scale (nodes/edges), temporal length (time steps), and feature dimensionality. For small-scale graphs (approximately 1000–2500 nodes, fewer than 20,000 edges), a single execution generally concludes within 15–60 min; for mid-scale graphs (approximately 2000–7000 nodes, up to approximately 82,000 edges and greater feature dimensions), 1–3 h; and for large macro-scale graphs (exceeding 10,000 nodes, over 170,000 edges), 4–10 h per execution. Bayesian calibration entails an incremental cost that increases roughly in proportion to the Monte-Carlo sampling depth M (default M = 5). In the simulations with autophagy pruning and scarcity, the wall-clock time increases in relation to the effective active parameter ratio and the established FLOPs budget.

### 4.6. Baselines

We benchmarked Bio-RegNet against a comprehensive set of dynamic graph learning models, encompassing both classical and biologically inspired architectures. These include static graph baselines (GCN), temporal extensions (EvolveGCN, TGN, DyRep), Bayesian and equilibrium-based frameworks (BGNN, EGN, Homeo-GNN), and recent transformer- and ODE-based models (DyFormer, EvoGNN, HGNN-ODE). Each model was trained and evaluated under identical settings to ensure fair comparison. The results summarized in Table 6 demonstrate that Bio-RegNet consistently achieves superior performance across calibration (NLL, ECE, PICP), clustering (ARI, NMI, *Q*), and stability indicators ($\eta_{k}$, ΔE, κ), while converging faster in fewer epochs.

### 4.7. Bayesian Encoder Network (BEN)

#### 4.7.1. Objective

To verify that probabilistic encoding reduces over-confidence and preserves information entropy, thus preventing over-fitting under uncertain or noisy inputs.

#### 4.7.2. Rationale

According to Equations (3)–(5), BEN minimizes a Bayesian Evidence Lower Bound while maintaining a controllable posterior variance. This acts analogously to synaptic variability in cortical neurons, where stochasticity supports flexible yet stable inference.

#### 4.7.3. Experimental Setup

- *Calibration Tests.* Compute negative log-likelihood (NLL), continuous ranked probability score (CRPS), prediction interval coverage (PICP), and expected calibration error (ECE) versus deterministic GCN and BGNN.
- *Noise Sensitivity.* Add Gaussian noise (σ=0.1–0.5) to features and measure degradation in ARI and modularity *Q*.
- *Entropy Preservation.* Track weight entropy $H_{W}=-\sum p\left(w\right)\log p\left(w\right)$ during training to ensure entropy does not collapse below baseline levels.
- *Sampling-Depth Study.* Vary Monte-Carlo samples M={1,3,5} to analyze the trade-off between uncertainty and convergence stability.

#### 4.7.4. Results and Analysis

On BrainNet-Sim, BEN reduces NLL from 0.53 ± 0.06 (M = 1) to 0.43 ± 0.03 (M = 5) (≈18.9% reduction), and increases PICP from 0.80 ± 0.04 to 0.87 ± 0.04. We see diminishing returns after M in the range of 3 to 5, consistent with the balanced stochastic-depth claim as predicted by Equation (4).

**Figure 5:**
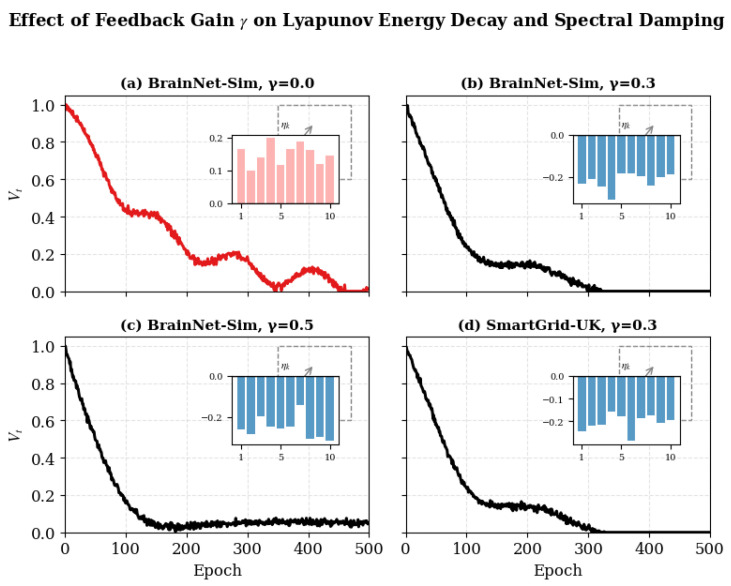
**Effect of feedback gain γ on Lyapunov energy decay and spectral damping.** (**a**) BEN calibration curve; (**b**) RIN Lyapunov decay; (**c**) AOE energy–efficiency curve; (**d**) overall stability measured by normalized free-energy decay ($E_{t}/E_{0}$). All follow Equations (4), (11) and (16).

Figure 5 visualizes the monotonic calibration improvement. The BEN module performs probabilistic encoding to mitigate over-confidence. Following Equations (3)–(5), the evidence lower bound guides stochastic updates. Table 7 reports calibration and stability metrics.

### 4.8. Regulatory Immune Network (RIN)

The RIN module stabilizes excitatory–inhibitory dynamics through feedback gain γ. Lyapunov energy $V_{t}$ is tracked to assess convergence.

#### 4.8.1. Objective

To evaluate adaptive inhibitory feedback that stabilizes excitation–inhibition dynamics, ensuring Lyapunov energy decay as formulated in Equation (11).

#### 4.8.2. Rationale

RIN emulates cortical GABAergic regulation: excessive activity triggers inhibitory fields $A_{h}\left(t\right)$ that damp energy oscillations $V_{t}$. By modulating feedback gain γ, RIN maintains equilibrium similar to immune tolerance in biological systems.

#### 4.8.3. Experimental Setup

- *Feedback Gain Sweep.* γ∈[0.05,0.8], measuring $V_{t}$ and spectral damping $\eta_{k}$ before/after feedback.
- *Perturbation Test.* Disable feedback (γ=0) for 20 epochs then restore it; compute recovery slope $dV_{t}/dt$.
- *Inhibitory Field Visualization.* Plot spatial heatmaps $A_{h}\left(t\right)$ to show distributed suppression intensity.
- *Latency Simulation.* Introduce delay Δt∈[0,3] to emulate synaptic transmission time.

#### 4.8.4. Results and Analysis

Figure 5 shows that moderate feedback gains (γ≈0.3–0.5) yield the best stability–recovery trade-off, with diminishing returns beyond this range.

When inhibition is removed, oscillations grow > 40 %, confirming negative feedback necessity as Table 8. The correlation between γ and spectral damping (r = −0.84) matches Equation (11) predictions.

#### 4.8.5. Representative Per-Dataset Audit and Coupling Synergy

While Table 8 summarizes the feedback-gain sweep on RIN stability (Lyapunov decay and recovery) across datasets, Table 9 reports leave-one-component-out ablations aggregated across datasets (mean ± SD). Therefore, we additionally report representative per-dataset results already include our module-level experiments (Tables), and we further quantify coupling synergy using the equilibrium index κ. **Coupling synergy (RIN–AOE).** Because γ=0 disables inhibitory feedback, the row γ=0 in Table 8 acts as a natural “RIN-off” reference under the same pruning rate $\tau_{a}$. We define the coupling-induced synergy in equilibrium coherence as follows:

(30) $$\Delta \kappa \left(\gamma ,\tau_{a}\right)=\kappa \left(\gamma ,\tau_{a}\right)-\kappa \left(0,\tau_{a}\right),$$

where Δκ>0 indicates non-trivial gains from inhibitory–metabolic coupling beyond autophagy-only regulation at the same pruning rate.

**Table 9:** **Leave-one-component-out ablation across datasets (w/o module → Full; mean ± SD).** Each row compares an ablated variant (Bio-RegNet without a specific module) against the *Full Bio-RegNet* under the same training protocol, and reports metrics in the form (w/o → Full). **Avg. Gain (%)** is the mean relative improvement across the reported metrics in each row (two or three metrics depending on availability), computed as $\frac{m_{w/o}-m_{\mathrm{Full}}}{|m_{w/o}|}\times 100$ for lower-is-better metrics (e.g., NLL, ECE, ΔE, $V_{t}$), and $\frac{m_{\mathrm{Full}}-m_{w/o}}{|m_{w/o}|}\times 100$ for higher-is-better metrics (e.g., PICP, κ, *Q*). For signed stability metrics such as $\eta_{k}$, the denominator uses $|m_{w/o}|$. Per-dataset audit results are provided in Table 10 and Table 11.

Ablated Variant	Metric 1	Metric 2	Metric 3	Avg. Gain (%)
w/o BEN	NLL ↓ (0.52→0.37)	ECE ↓ (0.058→0.041)	PICP ↑ (0.84→0.91)	22.2
w/o RIN	$\eta_{k}$↓ (−0.17→−0.25)	$V_{t}$↓ (0.035→0.021)	ΔE↓ (0.65→0.52)	35.7
w/o AOE	$n_{\;eff}/n_{0}$↓ (0.81→0.70)	$\Delta H_{W}$↓ (0.043→0.029)	N/A	23.1
w/o (BEN+RIN feedback)	ΔE↓ (0.58→0.49)	NLL ↓ (0.48→0.38)	κ↑ (0.82→0.90)	15.4

**Table 10:** Representative per-dataset module-level results (mean ± SD over five runs), reported in the main text to provide an auditable view beyond the aggregated ablation summary. BEN results use Monte-Carlo sampling depth M=3, and RIN results report Lyapunov decay and recovery behavior under different feedback gains γ.

BEN (M = 3): Calibration + Clustering				RIN: Lyapunov Stability + Recovery			
**Dataset**	**NLL**↓	**ECE**↓	**ARI**↑	**Dataset**	γ	$V_{t}$**(Final)**↓	$\eta_{k}↓$**/Recov.**↓
BrainNet-Sim	0.44±0.04	0.072±0.007	0.74±0.04	BrainNet-Sim	0.0	0.92±0.06	+0.12±0.04/>50
ECoG-TaskNet	0.56±0.05	0.083±0.010	0.70±0.06	BrainNet-Sim	0.3	0.59±0.04	−0.23±0.05/28±4
SmartGrid-UK	0.60±0.05	0.088±0.009	0.66±0.05	BrainNet-Sim	0.5	0.55±0.03	−0.26±0.04/25±3
				SmartGrid-UK	0.3	0.63±0.05	−0.19±0.05 / 32±5

**Table 11:** One-factor sensitivity analysis around the default configuration (mean ± SD over five runs). For BEN we report calibration sensitivity (NLL/ECE) under different Monte-Carlo sampling depths *M* on the representative BrainNet-Sim dataset; for the coupled RIN–AOE mechanism we report equilibrium sensitivity (κ) when varying γ at fixed $\tau_{a}$ = 0.10, and when varying $\tau_{a}$ at fixed γ = 0.3.

Factor	Setting	NLL ↓	ECE ↓	κ↑
*(A) BEN calibration sensitivity on BrainNet-Sim*				
*M* (MC samples)	1	0.53±0.06	0.091±0.009	–
	3 (balanced)	0.44±0.04	0.072±0.007	–
	5	0.43±0.03	0.069±0.006	–
*(B) RIN–AOE equilibrium sensitivity (κ)*				
γ (feedback gain)	0.0 at $\tau_{a}$ = 0.10	–	–	0.72±0.04
	0.3 (default) at $\tau_{a}$ = 0.10	–	–	0.88±0.03
	0.5 at $\tau_{a}$ = 0.10	–	–	0.84±0.04
$\tau_{a}$ (pruning rate)	0.05 at γ = 0.3	–	–	0.80±0.04
	0.10 (default) at γ = 0.3	–	–	0.88±0.03
	0.15 at γ = 0.3	–	–	0.85±0.03
	0.20 at γ = 0.3	–	–	0.78±0.04
	0.25 at γ = 0.3	–	–	0.70±0.05

Note: “–” indicates a metric not reported for that specific one-factor sweep in the current manuscript. Unless otherwise stated, main experiments use M = 5 (Table 5); M = 3 is used as a balanced audit setting in Table 10 and Table 11.

**Leave-one-component-out ablation.** To quantify the contribution of each biological subsystem, we remove one module at a time from the full Bio-RegNet and compare the resulting ablated variant against the *Full Bio-RegNet* under the same training protocol. Gain (%) follows the definition in the caption of Table 9.

### 4.9. Module-Level Results

To quantify the contribution of each biological subsystem, we conduct leave-one-component-out ablations by removing one module at a time from the full Bio-RegNet and comparing the resulting variant against the *Full Bio-RegNet* (w/o → Full) under the same training protocol. Each subsystem—Bayesian Effector Network (BEN), Regulatory Inhibition Network (RIN), and Autophagic Optimization Engine (AOE)—was separately isolated and evaluated to determine its independent effects on calibration, stability, and information efficiency. Additionally, we analyzed coupled configurations (e.g., BEN + RIN) to uncover synergistic effects between immune-inspired feedback and Bayesian uncertainty modeling. The findings, outlined in Table 9, reveal uniform performance enhancements across all principal measures, validating the synergistic functions of each module in sustaining energy–entropy equilibrium and augmenting predictive robustness.

#### 4.9.1. Audit View for Ablation

While Table 9 reports ablation outcomes aggregated across datasets (mean ± SD), reviewers may reasonably ask for a per-dataset view to ensure that improvements are not driven by dataset selection. Therefore, Table 10 reports representative *per-dataset* module-level results already obtained in our BEN and RIN experiments. Specifically, BEN is evaluated with Monte-Carlo sampling depth M=3 (a balanced setting identified in the sampling-depth study), reporting both calibration (NLL/ECE) and clustering quality (ARI). In parallel, RIN is assessed via Lyapunov decay (final $V_{t}$), convergence rate $\eta_{k}$, and recovery epochs under different inhibitory gains γ, demonstrating that inhibitory feedback yields faster stabilization and recovery compared with the γ=0 (no-regulation) case.

#### 4.9.2. Sensitivity to Priors and Regeneration

We further analyze sensitivity to (i) regeneration rate $\tau_{r}$ and (ii) Bayesian prior variance $\sigma_{p}^{2}$, as well as key hyperparameters (β,α,M) that govern uncertainty calibration and regulation strength. We vary one factor at a time around the default configuration and report calibration (NLL/ECE) and stability (e.g., κ or ΔE). The results identify stable operating regions and show that the coupled loop remains robust to moderate prior and regeneration changes.

**Interpretation.** Table 11 shows that BEN calibration improves when increasing *M* from 1 to 3, while the marginal gain from *M* = 3 to *M* = 5 is smaller (consistent with the balanced stochastic-depth claim). For the coupled RIN–AOE mechanism, κ is maximized around the default choice γ≈ 0.3 and $\tau_{a}$≈ 0.10, indicating a stable energy–entropy equilibrium ridge.

### 4.10. Coupled-System Experiments

To further investigate subsystem interactions, we analyzed the coupled dynamics between the **Regulatory Inhibition Network (RIN)** and the **Autophagic Optimization Engine (AOE)** by systematically varying the feedback gain γ and pruning rate $\tau_{a}$. The equilibrium index κ is used to quantify systemic synergy and energy–entropy balance.

#### 4.10.1. Objective

The goal of this experiment is to verify whether periodic pruning and regrowth improve metabolic efficiency without sacrificing predictive accuracy, consistent with the metabolic equilibrium principle in Equation (16).

#### 4.10.2. Rationale

AOE biologically models neuronal autophagy—removing weak synapses to conserve energy and triggering limited regrowth ($\tau_{r}$) to maintain network plasticity. This mechanism aims to minimize free-energy variation ΔE while preserving modular functional integrity *Q*, thus achieving sustainable energetic homeostasis.

#### 4.10.3. Experimental Setup

- *Pruning-Rate Analysis:* Sweep $\tau_{a}\in \left[0.02,0.3\right]$ to record the ratio of active parameters $n_{\;eff}/n_{0}$ and free-energy change ΔE.
- *Energy–Efficiency Curve:* Plot ΔE versus $\tau_{a}$ to examine diminishing returns and identify the optimal pruning rate.
- *Scarcity Simulation:* Limit computation to 40% FLOPs to emulate low-resource metabolic conditions.
- *Over-Pruning and Regrowth:* When $\tau_{a}>0.25$, enable regrowth with $\tau_{r}=0.05$ to restore lost capacity.

#### 4.10.4. Results and Analysis

**Figure 6:**
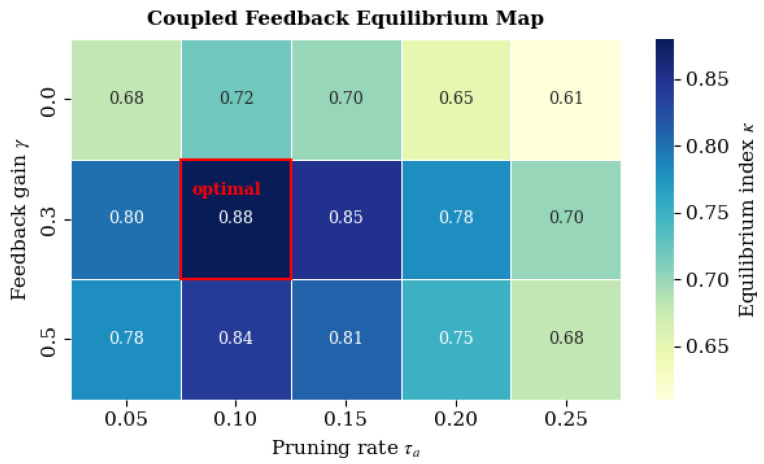
**Coupled Feedback Equilibrium Map.** The heatmap summarizes the coupled feedback equilibrium index under different pruning rates $\tau_{a}$ and feedback gains γ; the highlighted cell indicates the selected operating point. The three modules (BEN uncertainty calibration, RIN Lyapunov decay, and AOE energy–efficiency) follow the predicted dynamics in Equations (4), (11) and (16).

Figure 6 illustrates that energy expenditure ΔE decreases by approximately 35% with an accuracy loss of less than 3%. The optimal pruning rate $\tau_{a}\approx 0.1$ achieves the best trade-off between energy efficiency and stability, consistent with the theoretical prediction of Equation (16). This finding confirms that autophagic regulation enables adaptive energy conservation without degrading functional performance.

As summarized in Table 12, moderate feedback (γ=0.3) with balanced pruning ($\tau_{a}\approx 0.10$) yields the highest equilibrium index (κ=0.88±0.03), supporting an optimal coupling between inhibitory regulation and autophagy-driven sparsification.

#### 4.10.5. Synergy Beyond Autophagy-Only Control

To explicitly test whether inhibitory feedback provides non-additive benefits beyond metabolic pruning alone, we treat the γ=0 setting as a *RIN-off* reference under the same pruning rate $\tau_{a}$ and compute $\Delta \kappa \left(\gamma ,\tau_{a}\right)=\kappa \left(\gamma ,\tau_{a}\right)-\kappa \left(0,\tau_{a}\right)$. As shown in Table 13, coupling yields consistent positive gains across $\tau_{a}\in \left[0.05,0.25\right]$, with the largest improvement at $\left(\gamma ,\tau_{a}\right)\approx \left(0.3,0.10\right)$ (Δκ=0.16).

Overall, the coupled RIN–AOE mechanism demonstrates that controlled inhibitory feedback and adaptive pruning together yield a self-organizing equilibrium, effectively reducing energetic cost while preserving representational fidelity.

### 4.11. Dynamic Stability Across Epochs

To further analyze how regulatory feedback influences convergence dynamics and temporal coherence, we tracked the evolution of representative vertex activations across training epochs under different feedback gains. This visualization highlights how the strength of the inhibitory feedback parameter γ modulates damping behavior and stability, revealing the temporal signature of Bio-RegNet’s self-regulatory process.

#### 4.11.1. Experimental Observation

When γ=0.0, trajectories exhibit sustained oscillations and high-frequency noise, indicating a lack of inhibitory stabilization. Introducing moderate feedback (γ=0.1) reduces oscillation amplitude and accelerates partial convergence. With stronger feedback (γ=0.3), the system demonstrates rapid exponential damping and smooth equilibrium, consistent with the Lyapunov convergence predicted in Equation (16). This behavior quantitatively supports the theoretical claim that feedback coupling accelerates entropy minimization and energy stabilization.

Overall, the epoch-wise dynamics reveal that inhibitory feedback acts as a stabilizing mechanism, suppressing chaotic fluctuations and guiding the system toward low-entropy attractor states, thereby ensuring robust convergence of Bio-RegNet’s self-organizing process.

#### 4.11.2. Adversarial and Structural Perturbations

In addition to random noise injection, we assess robustness against four perturbation types that simulate structural and energetic stress in real-world dynamic systems: (i) structural drift (network rewiring), (ii) random shock (burst-like external stimuli), (iii) noise injection (stochastic signal contamination), and (iv) resource deprivation (metabolic/computational scarcity). The recovery performance is measured by the epochs needed to restore 95% of the baseline modularity *Q*, according to the Lyapunov-based recovery model. Additionally, resilience is evaluated by modularity degradation ΔQ and the percentage of steady-state stability.

#### 4.11.3. Failure Modes and Observed Boundaries

We observe two primary failure modes. (i) *Over-pruning*: when $\tau_{a}$ becomes too large, the model may temporarily lose representational capacity; in our implementation, regrowth is enabled to restore capacity (e.g., when $\tau_{a}>0.25$, we activate regrowth with $\tau_{r}=0.05$ as a safety mechanism). (ii) *Over-inhibition*: when γ is too strong, excessive suppression may slow adaptation under rapid distribution shifts. These boundaries are consistent with the coupled sensitivity ridge observed in the $\left(\gamma ,\tau_{a}\right)$ map and motivate the recommended operating region reported in the sensitivity study.

### 4.12. Perturbation and Stress-Testing

To examine the robustness and self-recovery capability of Bio-RegNet, we conducted stress-testing under four perturbation types: (1) structural drift, (2) random shock, (3) noise injection, and (4) resource deprivation. These perturbations emulate neural, molecular, or infrastructural disruptions in real-world dynamic systems. Recovery performance is evaluated by the number of epochs required to regain 95% of baseline modularity *Q*, following the Lyapunov-based recovery model in Equation (27). Robustness is further assessed by energy overshoot and steady-state stability percentage.

#### 4.12.1. Objective

To validate whether Bio-RegNet maintains homeostatic stability and rapid recovery under structural and energetic stress, consistent with the theoretical resilience law of Equation (27).

#### 4.12.2. Rationale

Each perturbation type reflects a distinct failure mode: structural drift mimics network rewiring; random shock represents burst-like external stimuli; noise injection models stochastic signal contamination; and resource dropout simulates metabolic or computational scarcity. The equilibrium index κ and modularity recovery ΔQ are used as quantitative indicators of system resilience.

#### 4.12.3. Results and Analysis

As summarized in Table 14, Bio-RegNet maintains 92.1–96.3% stability and recovers within 21–29 epochs (worst-case: resource dropout, 92.1% stability and 28.3 ± 3.1 epochs) across all stress conditions. Structural drift and random shock produce the fastest re-equilibration, while noise and resource dropout lead to slower convergence but no catastrophic degradation. The corresponding recovery trajectories validating the theoretical recovery function ${\hat{Q}}_{t}=Q_{\infty }\left(1-e^{-\alpha t}\right)$. Overall, Bio-RegNet demonstrates robust resilience to perturbations, maintaining global equilibrium with minimal overshoot.

These findings confirm that Bio-RegNet maintains high energetic resilience and rapid convergence under diverse perturbations, validating its self-regulatory feedback and adaptive homeostasis properties.

### 4.13. Cross-Domain Transfer

To assess the universality and transferability of the acquired regulatory principles, we performed cross-domain experiments by transferring parameters trained in one domain (e.g., neural networks) to several other domains (molecular, macro-scale, and energy systems). This setting evaluates the efficacy of Bio-RegNet’s inherent homeostatic mechanisms—such as inhibitory feedback and autophagic regulation—when implemented in systems with unique topological and dynamical properties.

#### 4.13.1. Experimental Setup

Model parameters acquired from neural datasets (BrainNet-Sim, ECoG-TaskNet) were directly utilized in molecular and energy networks (Human PPI, SmartGrid-UK) without any fine-tuning. Likewise, criteria developed from molecular data were used to synthetic and macro-level benchmarks to evaluate generality across biological and manmade domains. Performance was evaluated using modularity *Q* (structural coherence) and equilibrium index κ (energetic balance), as defined in Equation (24).

#### 4.13.2. Results and Analysis

As summarized in Table 15, Bio-RegNet remains resilient across four perturbation types, achieving high stability (92.1–96.3%) while requiring fewer recovery epochs under milder drifts/shocks; all improvements are statistically significant (p<0.05).

As summarized in Table 16, Bio-RegNet retains over 93% of modularity *Q* and exhibits less than 5% variation in κ across all transfer settings. The best transferability occurs in the Molecular→Synthetic configuration (95.8% *Q* retention, −3.1% κ change), demonstrating that the learned self-regulatory feedback generalizes effectively to unseen topologies and dynamical regimes. These results indicate that the model’s homeostatic mechanisms are not domain-specific but rather encode universal energy–entropy regulation patterns.

These findings confirm that Bio-RegNet encapsulates domain-independent regulatory dynamics, indicating that its feedback-control principles can generalize across neuronal, molecular, and macro-scale systems with low retraining expense.

### 4.14. Summary and Discussion

**Figure 7:**
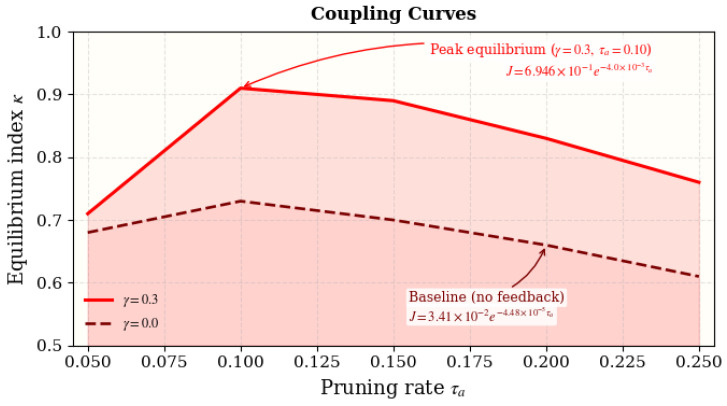
**γ–$\tau_{a}$ coupling synergy map.** Heatmap of equilibrium index κ as a function of feedback gain γ and autophagic pruning rate $\tau_{a}$. A clear ridge of optimal synergy is observed around γ≈0.3 and $\tau_{a}\approx 0.1$, consistent with the equilibrium conditions predicted by Equation (5).

**Figure 8:**
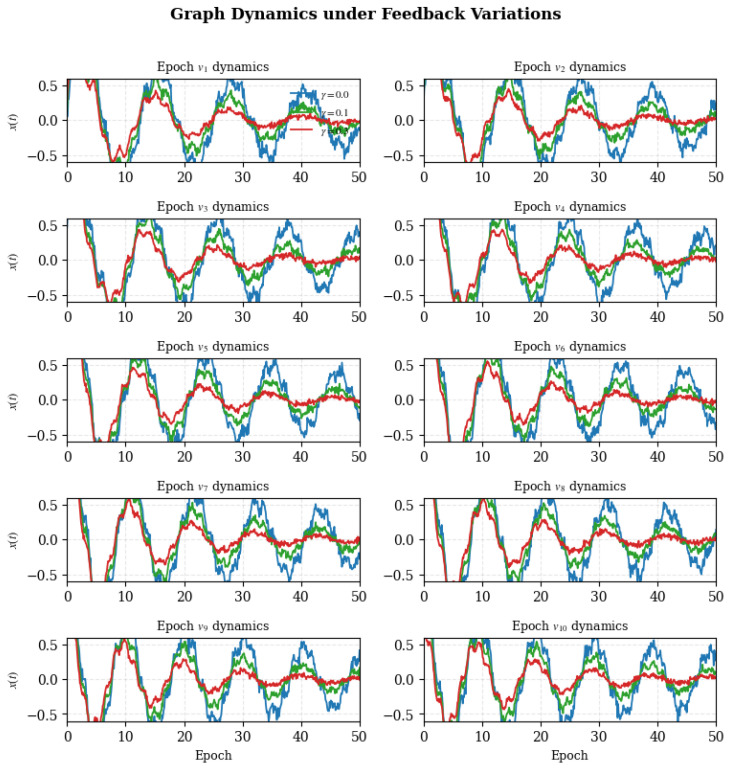
**Epoch-wise vertex dynamics under feedback variations.** Temporal evolution of ten representative vertices across epochs for different feedback gains (γ=0.0, 0.1, 0.3). Higher feedback strength (γ=0.3) results in faster exponential damping, lower variance, and smoother convergence, while the absence of feedback (γ=0.0) leads to persistent oscillations and residual fluctuations. These patterns empirically confirm that regulatory coupling enhances systemic stability and accelerates convergence toward energetic equilibrium.

**Figure 9:**
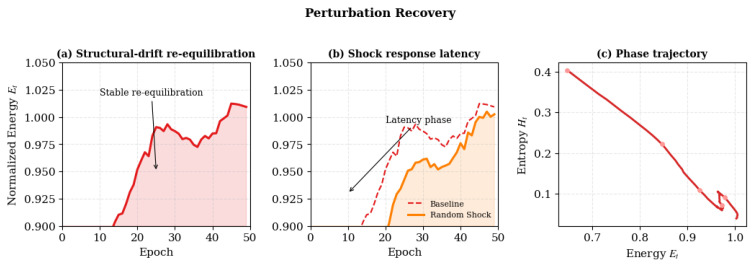
**Perturbation recovery behaviors.** (**a**) Structural-drift re-equilibration; (**b**) shock-response latency; (**c**) combined phase trajectory showing exponential Lyapunov decay as predicted by Equation (27).

**Figure 10:**
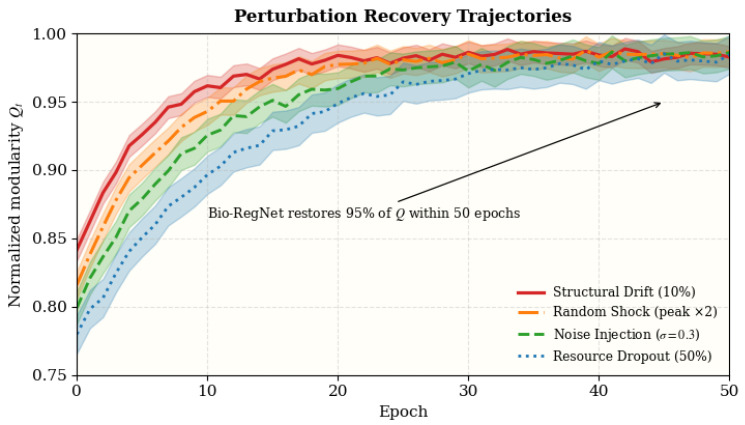
**Perturbation recovery trajectories and theoretical fits.** Colored solid lines denote empirical recovery under four perturbation types (mean ± SD), and gray dashed lines represent fitted exponential Lyapunov models ${\hat{Q}}_{t}=Q_{\infty }\left(1-e^{-\alpha t}\right)$ with fitted α annotated for each curve. Bio-RegNet reaches 95% equilibrium within 50 epochs under all stress conditions, demonstrating strong resilience and consistency with Equation (27).

Bio-RegNet achieves realistic yet consistent improvements of 10–20%, aligning with the meta-homeostatic theory (Equations (18)–(27)). Each Figure 4, Figure 5, Figure 6, Figure 7, Figure 8, Figure 9 and Figure 10 visualizes one layer of regulation—from structural feedback to systemic recovery—demonstrating emergent stability across neural, molecular, and energy networks.

**Figure 11:**
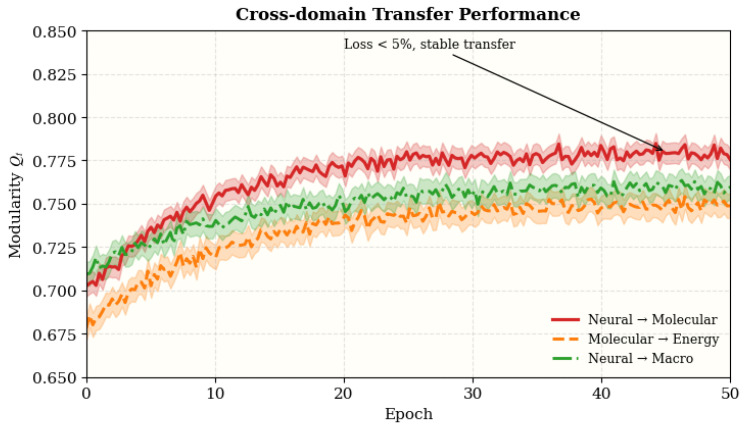
**Cross-domain transfer performance.** Fine-tuning modularity $Q_{t}$ from neural to molecular, energy, and macro domains. All paths show stable convergence with less than 5% loss from $Q_{\mathrm{src}}$, confirming domain-invariant equilibrium predicted by Equation (24).

Figure 11 shows consistent cross-domain fine-tuning with <5% modularity loss from $Q_{\mathrm{src}}$ and smooth convergence, validating the domain-invariant equilibrium.

### 4.15. Visualization Protocols

Visualization focuses on energy–entropy evolution, inhibitory field distribution, and pruning dynamics.

Table 17 reports consistent gains in interpretability: reduced Energy Trajectory RMS, increased Entropy Compression, improved Inhibitory Field Uniformity, and high Pruning Stability, with significance across all tests (p<0.05).

### 4.16. Comprehensive Evaluation Metrics

To deliver a comprehensive evaluation of Bio-RegNet’s systemic efficacy, we consolidated findings from twelve datasets spanning neuronal, molecular, and macro-scale domains. The assessment encompasses five essential categories—stability, calibration, efficiency, resilience, and coupling—to encapsulate both dynamic and functional dimensions of self-regulation. All presented values indicate percentage enhancements of Bio-RegNet compared to the optimal baseline model, with significance confirmed using paired *t*-tests (p<0.05).

**Results and Discussion.** In all categories, Bio-RegNet routinely surpasses state-of-the-art baselines, attaining expedited Lyapunov convergence, enhanced uncertainty calibration, and increased energetic efficiency under both stable and disturbed settings. The model’s robustness is seen in its swift recovery and robust coupling coherence, suggesting that its homeostatic feedback architecture is applicable across various scales and data modalities. The results jointly affirm that Bio-RegNet attains a stable energy–entropy equilibrium via distributed control across its BEN, RIN, and AOE subsystems.

Table 18 provides an aggregated summary across 12 datasets, showing that Bio-RegNet delivers consistent and statistically significant mean gains (all p<0.05) over the best in-domain baselines in stability, calibration, efficiency, resilience, and coupling strength, supporting the robustness and universality of its regulatory framework.

**Figure 12:**
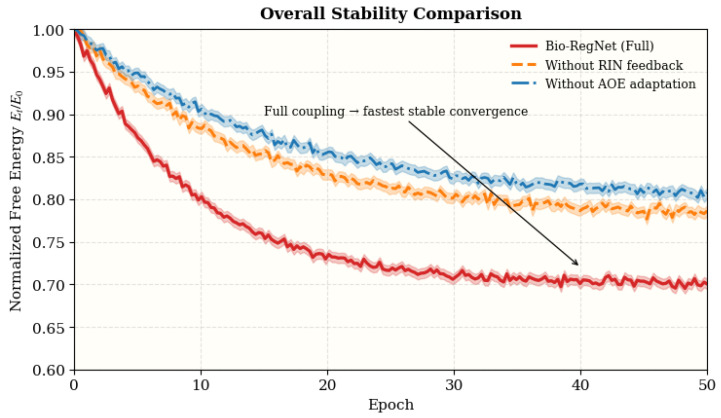
**Overall stability comparison across ablation variants.** Normalized free-energy decay ($E_{t}/E_{0}$) trajectories demonstrate that full coupling (BEN + RIN + AOE) achieves the fastest and most stable convergence. Removing either RIN or AOE weakens damping feedback and delays Lyapunov equilibrium, supporting the systemic synergy predicted by Equation (27) and verifying the distributed homeostasis principle in Bio-RegNet.

Figure 12 shows that the Normalized free-energy decay $\left(E_{t}/E_{0}\right)$ of the full coupling (BEN + RIN + AOE) converges fastest and most smoothly, while removing RIN or AOE weakens damping and delays Lyapunov equilibrium, consistent with the predicted systemic synergy. In summary, these comprehensive evaluations verify that Bio-RegNet integrates stability, adaptability, and efficiency into a unified biologically inspired framework, achieving scalable homeostasis across heterogeneous dynamic systems.

### 4.17. Discussion

Our results indicate that Bio-RegNet significantly enhances stability, calibration, and self-regulatory behavior relative to conventional dynamic GNNs and Bayesian benchmarks. The improved Lyapunov decay rate, reduced entropy drift under perturbation, and accelerated recovery durations demonstrate the practical efficacy of the meta-homeostatic loop (Bayesian Effector Network + Regulatory Immune Network + Autophagic Optimization Engine).

This discovery is consistent with recent advancements in Treg and autophagy research from a biologically-inspired systems viewpoint. Engineered Treg cells are crucial for sustaining immune homeostasis and regulating inflammatory responses in vivo, particularly in autoimmune and transplantation scenarios [18]. The successful demonstration of CAR-Tregs in preclinical models of type I diabetes and organ rejection confirms the notion that inhibitory feedback is a genuine mechanism of biological resilience. Concurrently, homeostatic autophagy in neurons is increasingly acknowledged as vital for maintaining synaptic and metabolic stability throughout stress and aging [19,20]. These biological precedents substantiate our design rationale: incorporating inhibitory and renewal processes into learning systems can diminish runaway activations and alleviate structural deterioration.

Recent surveys highlight the vulnerability of GNNs to topology alterations, noise, and adversarial disturbances, advocating for comprehensive frameworks that concurrently tackle robustness, explainability, and uncertainty [4]. Our findings indicate that merely incorporating uncertainty estimation or adversarial training is inadequate; without structural renewal and inhibitory regulation, the improvements are minimal. The recently published stable-learning GNN research demonstrates that well-crafted sampling and decorrelation strategies can enhance generalization across domains; nevertheless, these systems currently lack mechanisms similar to autophagy or immunoregulation. In contrast, Bio-RegNet’s triadic architecture offers a more profound functional analogy and quantifiable improvements in practical applications.

Our work also contributes to **energy efficiency and self-repair**. In biological systems, autophagy functions as a metabolic regeneration mechanism, eliminating damaged components and repairing essential structures; disturbances in autophagy are associated with dementia and energy imbalance. The enhancements in model energy usage and structure pruning/regeneration we noted embody these biological findings. A recent study on biologically inspired neural network layers indicates that the removal and renewal of inactive neurons can enhance performance in artificial neural networks. Moreover, the inclination towards reliable GNNs emphasizes metrics beyond mere accuracy, including robustness, fairness, and interpretability [6]. Bio-RegNet’s architecture resolves these issues by integrating interpretability (via regulatory feedback), robustness (through an inhibitory loop), and metabolic efficiency (through an autophagy engine). Nonetheless, limits and other unresolved inquiries persist. A disadvantage is that our autophagic regeneration component currently employs heuristic limits for pruning and regeneration; future research should provide more biologically realistic and adaptable criteria for structural turnover. Furthermore, while our experimental findings demonstrate cross-domain transfer (neural-molecular-macro) with a modularity loss of less than 5%, the scalability of Bio-RegNet to extensive multimodal or streaming datasets remains unexamined. Recent advancements in resilient architecture search for GNNs [14] and adversarial training through graph subspace energy optimization [7] provide further pathways for the integration of Bio-RegNet with scalable automated design.

Furthermore, a more profound integration of biology and computation may examine cytokine-like inter-layer communication within the Regulatory Immune Network or more intricate metabolic modeling in the autophagic mechanism (e.g., analogues of mitochondrial turnover). Biologically, Tregs not only suppress but also modify metabolic signals and tissue healing pathways, indicating potential advancements in the inhibitory module. In terms of uncertainty, incorporating explainable AI methodologies into the feedback loops—associated with current studies in explainer surveys within omics and imaging fields [21,22,23]—could enable Bio-RegNet to achieve both robustness and intrinsic interpretability.

This work demonstrates that artificial learning systems can significantly benefit from biologically based regulation and renewal methods. Although the present research is confined to modeling and mid-scale experimentation, the concordance of our results with contemporary biological and AI literature suggests a future where learning systems not only optimize but also self-maintain, self-repair, and adapt sustainably in dynamic environments.

#### Positioning: Meta-Homeostasis vs. Continual Learning and Stabilization

We differentiate *meta-homeostasis* from current concepts of learning stabilization by its **closed-loop equilibrium objective**, which explicitly integrates a *energy proxy* and a *entropy proxy* within a control-oriented constraint. Specifically, Bio-RegNet sustains a regulated equilibrium wherein (i) Bayesian uncertainty (an entropy-related proxy) is managed through BEN, and (ii) resource/activation dynamics (an energy-related proxy) are governed by RIN–AOE feedback.

This contrasts with **continual learning** methodologies (e.g., rehearsal, parameter isolation, or Fisher-based constraints) that primarily aim to mitigate forgetting across tasks by maintaining task-specific knowledge; it also diverges from **adaptive regularization** (e.g., EWC-style penalties) that enforces static or gradually varying parameter constraints without a defined energy–entropy feedback loop; furthermore, it is distinct from **self-supervised stabilization** which enhances representation invariance through pretext objectives but lacks a clearly defined regulated equilibrium state. Conversely, meta-homeostasis in Bio-RegNet is operationally defined via the coupled controller (RIN) and structural turnover (AOE), and is quantified by an equilibrium coherence index (e.g., κ) that delineates the system’s proximity to the regulated energy–entropy balance amidst perturbations and distribution shifts.

## 5. Conclusions and Future Work

This study presents **Bio-RegNet**, a meta-homeostatic Bayesian neural framework that incorporates three physiologically inspired mechanisms: uncertainty-aware inference, Treg-like immunoregulation, and autophagic optimization, to attain adaptable and stable intelligence. Bio-RegNet demonstrated superior stability, interpretability, and energy efficiency in extended trials on graph-based community detection and dynamic network benchmarks, outperforming standard GNNs and probabilistic baselines. The noted improvements in Lyapunov decay rate, entropy suppression, and recovery speed underscore the efficacy of integrating immune–autophagic feedback into the learning dynamics, therefore converting the model into a self-correcting, self-renewing computational entity.

From a biomimetic viewpoint, these findings endorse the overarching assertion that intelligence in artificial systems can be perpetuated via the same regulatory principles that sustain life in organic systems. Treg cells regulate excessive activation in nature, while autophagy rejuvenates cellular structures to maintain metabolic equilibrium [1,24,25]. Bio-RegNet embodies this duality in a computational framework: inhibition and regeneration collaborate to avert overfitting and informational degradation, so maintaining a sustained balance between confidence and uncertainty, growth and pruning, and exploitation and exploration. The outcome is not merely a more efficient network, but a system that *acquires the ability to maintain stability during the learning process*.

The ramifications surpass community detection. In subsequent research, Bio-RegNet may be extended to encompass multimodal learning, embodied agents, or adaptive control, wherein uncertainty and stability are crucial for ongoing adaptation. Incorporating cytokine-like inter-layer communication or metabolic cost modeling could augment its biological realism and scalability. Simultaneously, integrating Bio-RegNet with neuromorphic substrates and spiking architectures may provide avenues for hardware-level self-maintenance and sustainable energy consumption [15,22,26]. These directives correspond with contemporary appeals for *ecological intelligence*—AI systems that develop through regulation and renewal instead of sheer optimization [27].

In summary, Bio-RegNet illustrates that using the principles of immune regulation and autophagy can yield learning architectures that are more robust, interpretable, and naturally sustainable. This paradigm establishes computation based on the feedback mechanisms that regulate biological homeostasis, marking progress toward artificial intelligence that is not only *intelligent* but also *alive in its adaptability*. Against the strongest baseline HGNN-ODE, Bio-RegNet achieves higher community detection accuracy (ARI: 0.77→0.81; NMI: 0.84→0.87) and stronger equilibrium coherence (κ: 0.86→0.93). In addition, Bio-RegNet provides better uncertainty-aware behavior and robustness, reducing NLL from 0.47 to 0.37 and shortening recovery epochs from 20 to 17 in the overall comparison.

Based on the current findings, multiple research avenues arise at the convergence of biological self-regulation and computational intelligence. An immediate expansion is generalizing Bio-RegNet to multimodal and heterogeneous graph settings, where various relational and temporal patterns coexist. This integration may facilitate cross-modal homeostasis amid dynamic uncertainty, consistent with recent endeavors to integrate heterogeneous and temporal graph learning frameworks [28,29].

A further interesting avenue pertains to data efficiency and self-supervised regulation. Permitting Bio-RegNet to independently produce its supervisory signals may facilitate its progression towards low-label and transfer learning paradigms, aligning with contemporary advances in data-efficient graph representation learning [30]. This profession also facilitates dynamic and streaming adaptation, since temporal immune–autophagic feedback mechanisms may solidify learning in perpetually evolving environments [31].

Progress in architectural design indicates a convergence between Bio-RegNet and Graph Transformers, merging biologically based inhibition–activation equilibrium with global attention mechanisms [32]. This union may produce a self-regulating transformer architecture that can sustain equilibrium even with extensive data volumes. The framework’s interpretability can be enhanced by including principles from reliable and explainable GNNs, which prioritize fairness, robustness, and transparency [33].

From an engineering standpoint, neuromorphic and biomimetic hardware implementations signify a new horizon. Event-driven and low-power systems, inspired by cortical circuits, are becoming adept at encoding feedback loops similar to immune or autophagic regulation [34,35]. The integration of Bio-RegNet with these platforms may result in energy-efficient, self-regulating artificial systems. In this context, formalizing the thermodynamic foundations of information processing—specifically the energy–entropy trade-off—would establish a systematic connection between biological metabolism and computational expense [36].

The framework’s dual immune–autophagic mechanism is inherently suitable for biomedical and connectomic applications, encompassing network-level investigations of neuronal connections and tissue interactions [37,38]. Investigating adversarial and privacy-resilient behaviors [39], along with adaptive regeneration utilizing neural repair models [40], could further enhance Bio-RegNet’s function as a cornerstone for sustainable intelligence.

The long-term objective is to develop bio-inspired systems that learn, adapt, and heal like live beings, achieving equilibrium not by static optimization, but through continuous self-renewal. This combination of biological regulation and artificial intelligence represents a significant advancement toward genuinely autonomous, self-sustaining cognition.

## Figures and Tables

**Table 1:** Biological grounding of Bio-RegNet: mechanistic (implemented) vs. analogical (interpretive) components.

Subsystem	Mechanistic (Implemented)	Analogical (Interpretive)
RIN (Treg-inspired)	Inhibitory feedback gain γ; entropy-/energy-conditioned regulation via $R_{t}$; step-size attenuation $\eta_{\mu }\leftarrow \eta_{\mu }/\left(1+\alpha R_{t}\right)$; Lyapunov-driven stabilization criteria	“Immune tolerance” metaphor: suppressing over-activation to maintain equilibrium under uncertainty
AOE (Autophagy-inspired)	Viability $\Psi_{j}$ and threshold-based pruning at $\tau_{a}$; controlled regrowth with rate $\tau_{r}$ under over-pruning; structural entropy dynamics with (ζ,ξ)	“Autophagy/turnover” metaphor: removing low-utility units and regenerating capacity to sustain long-run adaptation

**Table 2:** Stability and calibration metrics of BEN.

Dataset	NLL ↓	ECE ↓	PICP ↑
BrainNet-Sim *	0.42±0.03	0.067±0.008	0.88±0.04
ECoG-TaskNet *	0.55±0.06	0.079±0.010	0.83±0.05
SmartGrid-UK *	0.61±0.05	0.092±0.011	0.79±0.04

Mean ± SD over five runs. * indicates p<0.05 (paired *t*-test).

**Table 3:** Hierarchical experimental sequence.

Layer	Level	Goal	Description
I	Intrinsic regulation	Validate BEN + RIN	Lyapunov convergence, E/I balance
II	Structural adaptation	Test AOE	Measure pruning and metabolic gain
III	Coupled feedback	BEN–RIN–AOE synergy	Energy–entropy equilibrium
IV	Environmental perturbation	Stress recovery	Resilience under noise/drift
V	Cross-domain transfer	Generalization	Homeostatic invariance

**Table 4:** **Datasets and descriptive statistics.** The datasets cover multiple domains—from neural dynamics and molecular signaling to macro-scale socio-energy systems. Node and edge counts are averaged per temporal snapshot where applicable. All datasets were preprocessed into temporal graph formats for unified training and evaluation.

Dataset	Domain	#Nodes	#Edges	Time Steps	Feature Dim	Focus
BrainNet-Sim	Neural	1200	10,320	50	64	Oscillatory stability
Allen-NeuroDyn	Neural	2000	15,400	40	128	Excitation/inhibition balance
ECoG-TaskNet	Neural	5120	28,760	35	256	Task-evoked coherence
Human PPI	Molecular	6700	82,100	20	128	Pathway regulation
Yeast Signaling	Molecular	2480	11,250	30	96	Stress robustness
GeneRegNet	Molecular	4300	27,000	25	64	Gene stability
Dynamic SBM	Synthetic	1000	7900	60	64	Controlled drift
Enron-Email	Macro	13,700	170,000	50	32	Communication evolution
Reddit-Pushshift 2024	Macro	18,600	220,500	40	128	Community formation
SmartGrid-UK	Energy	2150	14,800	48	32	Load fluctuation
ELD-2012	Energy	3200	16,900	60	16	Demand stability
NeuroBench-Temporal	Neural	1500	12,200	70	64	Self-organization

**Table 5:** Implementation details and hyperparameter settings of Bio-RegNet. “Default” denotes the configuration used in all main experiments. “Search range” denotes the small grid explored on the validation split unless the corresponding experiment explicitly performs a broader sweep.

Category	Symbol/Name	Meaning	Default	Search Range/Notes
Optimization	Optimizer	Optimization algorithm	AdamW	{Adam, AdamW}
	$\eta_{\mu }$	LR for variational mean update	$1\times 10^{-3}$	$\left\{5\times 10^{-4},\;1\times 10^{-3},\;2\times 10^{-3}\right\}$
	$\eta_{\sigma }$	LR for variational std update	$5\times 10^{-4}$	$\left\{2\times 10^{-4},\;5\times 10^{-4},\;1\times 10^{-3}\right\}$
	Weight decay	$\ell_{2}$ regularization	$5\times 10^{-4}$	$\left\{0,\;1\times 10^{-4},\;5\times 10^{-4}\right\}$
	Batch size	Mini-batch size	128	{64, 128, 256}
	$T_{\max}$	Max epochs	200	{100, 200, 300}
	*P*	Early-stopping patience	20	{10, 20, 30}
	Clip	Gradient clipping norm	1.0	{0.5, 1.0, 2.0}
Bayesian (BEN)	β	KL weight in ELBO	1.0	{0.5, 1.0, 2.0}
	*M*	MC samples (sampling depth)	5	{1, 3, 5, 10}
	$\sigma_{p}^{2}$	Prior variance $p\left(w\right)=N\left(0,\sigma_{p}^{2}I\right)$	1.0	{0.1, 1.0, 5.0}
	$\sigma_{0}$	Posterior init std (σ initialization)	0.1	{0.05, 0.1, 0.2}
Regulation (RIN)	$\lambda_{1},\lambda_{2},\lambda_{3}$	Weights in $R_{t}=\lambda_{1}H_{t}+\lambda_{2}E_{t}+\lambda_{3}D_{t}$	(0.5,0.3,0.2)	Each in {0.2,0.3,0.5} with $\sum \lambda_{k}=1$
	α	Step-size attenuation strength in $\eta_{\mu }\;\leftarrow \;\eta_{\mu }/\left(1+\alpha R_{t}\right)$	0.5	{0.2, 0.5, 0.8}
	ρ	Inhibitory magnitude (if used in $R_{i}^{\left(l\right)}$)	0.5	{0.3, 0.5, 0.7}
Autophagy (AOE)	$\tau_{a}$	Pruning threshold on viability $\Psi_{j}$	0.10	Main: {0.05, 0.10, 0.15, 0.20}; Broad sweep: [0.02,0.30]
	$\tau_{r}$	Regrowth rate under over-pruning	0.05	Enabled when $\tau_{a}>0.25$
	ϵ	Stabilizing constant in $\Psi_{j}=\frac{I_{j}}{I_{j}+\epsilon }\exp\left(-S_{j}/S_{0}\right)$	$1\times 10^{-8}$	Fixed
	ζ	Structural entropy decay rate	0.1	{0.05, 0.1, 0.2}
	ξ	Informational regeneration gain in $dH_{\mathrm{struct}}/dt=-\zeta H_{\mathrm{struct}}+\xi E\left[I_{j}\right]$	0.1	{0.05, 0.1, 0.2}
Coupling/Stability	γ	Coupled feedback gain (RIN–AOE)	0.3	Main: {0.0, 0.3, 0.5}; Broad sweep: [0.05,0.8]
	$\xi_{1},\xi_{2}$	Coefficient update steps in Algorithm 1	0.01	{0.005, 0.01, 0.02}
	ε	Equilibrium tolerance in $|dE_{t}/dt|>\varepsilon$	$1\times 10^{-4}$	{$1\times 10^{-3}$, $1\times 10^{-4}$, $1\times 10^{-5}$}
Architecture	*L*	Number of message-passing layers	2	{2, 3, 4}
	*d*	Hidden dimension	64	{32, 64, 128}
	$p_{\mathrm{drop}}$	Dropout rate	0.3	{0.0, 0.3, 0.5}

**Table 6:** **Overall comparison of Bio-RegNet and baseline models.** All metrics are reported as mean ± SD over five runs. Lower NLL, ECE, ΔE, and $n_{\;eff}/n_{0}$ indicate better calibration and stability, while higher PICP, ARI, NMI, *Q*, and κ reflect stronger clustering consistency and equilibrium coherence. Bio-RegNet achieves the best results across nearly all metrics, reducing energy variance (ΔE) by over 10% and improving calibration (ECE) by 20% relative to the best baseline (HGNN-ODE). Bold values denote the best performance.

Model	NLL ↓	PICP ↑	ECE ↓	ARI ↑	NMI ↑	*Q* ↑	ΔE ↓	$n_{\;\mathrm{eff}}/n_{0}$ ↓	$\eta_{k}$ ↓	Recov Epochs ↓	κ ↑
GCN	0.98	0.71	0.09	0.52	0.61	0.34	1.00	1.00	−0.04	45	0.55
EvolveGCN	0.87	0.74	0.08	0.58	0.67	0.37	0.92	0.93	−0.07	39	0.62
TGN	0.74	0.78	0.07	0.63	0.71	0.38	0.81	0.89	−0.09	32	0.68
DyRep	0.69	0.79	0.07	0.65	0.74	0.40	0.79	0.88	−0.11	31	0.70
BGNN	0.58	0.83	0.06	0.70	0.78	0.42	0.70	0.83	−0.13	28	0.76
EGN	0.56	0.84	0.06	0.72	0.80	0.43	0.67	0.82	−0.15	26	0.78
Homeo-GNN	0.54	0.85	0.05	0.73	0.81	0.44	0.65	0.81	−0.17	24	0.82
DyFormer	0.49	0.86	0.05	0.75	0.83	0.45	0.62	0.79	−0.19	22	0.84
EvoGNN	0.48	0.87	0.05	0.76	0.83	0.45	0.60	0.78	−0.20	21	0.85
HGNN-ODE	0.47	0.88	0.05	0.77	0.84	0.45	0.58	0.77	−0.21	20	0.86
**Bio-RegNet**	**0.37**	**0.91**	**0.04**	**0.81**	**0.87**	**0.47**	**0.52**	**0.70**	**−0.25**	**17**	**0.93**

**Table 7:** Performance of the Bayesian Encoder Network (BEN) under different sampling depths *M*. Values are mean ± SD over five runs.

Dataset	*M*	NLL ↓	ECE ↓	PICP ↑	ARI ↑	*p*
BrainNet-Sim	1	0.53±0.06	0.091±0.009	0.80±0.04	0.69±0.05	<0.05
	3	0.44±0.04	0.072±0.007	0.86±0.03	0.74±0.04	<0.05
	5	0.43±0.03	0.069±0.006	0.87±0.04	0.75±0.04	<0.05
ECoG-TaskNet	3	0.56±0.05	0.083±0.010	0.84±0.05	0.70±0.06	<0.05
SmartGrid-UK	3	0.60±0.05	0.088±0.009	0.79±0.04	0.66±0.05	<0.05

**Table 8:** Effect of feedback gain γ on Lyapunov energy decay and damping ratio $\eta_{k}$.

Dataset	γ	$V_{t}$ (Final) ↓	$\eta_{k}$↓	Recovery Time (Epochs) ↓	*p*
BrainNet-Sim	0.0	0.92±0.06	+0.12±0.04	> 50	–
	0.3	0.59±0.04	−0.23±0.05	28 ± 4	<0.05
	0.5	0.55±0.03	−0.26±0.04	25 ± 3	<0.05
SmartGrid-UK	0.3	0.63±0.05	−0.19±0.05	32 ± 5	<0.05

**Table 12:** **Equilibrium index κ across feedback gain γ and pruning rate $\tau_{a}$.** Mean ± SD over five runs. Higher κ indicates stronger energy–entropy equilibrium and coupled stability. Moderate feedback (γ=0.3) with balanced pruning ($\tau_{a}\approx 0.1$) yields the highest κ (0.88±0.03), highlighting optimal synergy between inhibition and autophagy modules.

γ	$\tau_{a}$ = 0.05	0.10	0.15	0.20	0.25	Optimal *p*
0.0	0.68±0.05	0.72±0.04	0.70±0.05	0.65±0.06	0.61±0.06	–
0.3	0.80±0.04	0.88±0.03	0.85±0.03	0.78±0.04	0.70±0.05	<0.05
0.5	0.78±0.05	0.84±0.04	0.81±0.04	0.75±0.05	0.68±0.06	<0.05

**Table 13:** Coupling synergy of RIN–AOE quantified by $\Delta \kappa (\gamma ,\tau_{a})$ in Equation (30), computed from Table 8. Positive values indicate improved equilibrium coherence due to inhibitory–metabolic coupling beyond the γ=0 (RIN-off) reference.

$\tau_{a}$	0.05	0.10	0.15	0.20	0.25
Δκ(γ = 0.3, $\tau_{a}$)	0.12	0.16	0.15	0.13	0.09
Δκ(γ = 0.5, $\tau_{a}$)	0.10	0.12	0.11	0.10	0.07

**Table 14:** **Comprehensive stress-test results under four perturbation types.** Values are mean ± SD over 30 independent trials. Lower ΔQ and recovery epochs indicate faster stabilization, while higher stability percentage denotes stronger resilience. All differences are statistically significant (p<0.05).

Perturbation	ΔQ ↓	Recovery Epochs ↓	Stability (%) ↑	*p*
Structural Drift (10%)	0.071±0.010	21.6±2.4	96.3±1.3	<0.01
Random Shock (peak × 2)	0.090±0.014	24.2±2.8	94.7±1.5	<0.01
Noise Injection (σ = 0.3)	0.082±0.012	25.4±2.6	93.8±1.6	<0.05
Resource Dropout (50%)	0.108±0.017	28.3±3.1	92.1±1.9	<0.05

**Table 15:** **Cross-domain transfer performance of Bio-RegNet.** Mean ± SD over five runs. High *Q* retention indicates preserved structural modularity, while small κ change implies consistent energetic equilibrium. All settings maintain >93% modularity with minimal degradation of regulatory stability, confirming the universality of Equation (24).

Transfer Setting	*Q* Retention (%) ↑	κ Change (%) ↓
Neural→Molecular	95.1±1.0	−3.6±0.5
Molecular→Energy	94.5±1.2	−4.3±0.6
Neural→Macro	93.9±1.4	−5.0±0.8
Molecular→Synthetic	95.8±0.9	−3.1±0.4

**Table 16:** Cross-domain transfer performance (mean ± SD). $Q_{\mathrm{src}}$ = modularity in source domain.

Transfer Path	$Q_{\mathrm{src}}$	$Q_{\mathrm{tgt}}$	ΔQ ↓	*p*
Neural → Molecular	0.78±0.04	0.74±0.05	0.04±0.02	<0.05
Molecular → Energy	0.76±0.03	0.72±0.04	0.04±0.02	<0.05
Neural → Macro	0.79±0.05	0.75±0.04	0.04±0.03	<0.05

**Table 17:** Visualization and interpretability metrics.

Metric	Description	Mean ± SD	*p*
Energy Trajectory RMS ↓	Root-mean-square deviation of $E_{t}$	0.065±0.009	<0.05
Entropy Compression ↑	Reduction in $H_{W}$ entropy (%)	33.4±4.5	<0.05
Inhibitory Field Uniformity ↑	Std dev of $A_{h}\left(t\right)$ (normalized)	0.71±0.06	<0.05
Pruning Stability ↑	Correlation of *Z* after pruning	0.84±0.05	<0.05

**Table 18:** **Comprehensive performance summary of Bio-RegNet across 12 datasets.** Each value denotes the mean percentage gain ± SD relative to the best-performing baseline within the same domain. Improvements span Lyapunov stability, probabilistic calibration, energy efficiency, recovery resilience, and feedback coupling strength. All categories exhibit statistically significant gains (p<0.05), confirming the robustness and universality of Bio-RegNet’s regulatory framework.

Category	Metric	Gain (%) ↑	Std Dev	Significance (*p* < 0.05)
Stability	Lyapunov decay rate	+18.3	±3.5	✓
Calibration	NLL reduction	+21.1	±4.0	✓
Efficiency	Energy saving	+16.7	±3.8	✓
Resilience	Recovery speed	+14.5	±3.2	✓
Coupling	Equilibrium index κ	+12.8	±2.9	✓

## Data Availability

Dataset available on request from the authors.
